# Deletion of *Ptpn2* in B cells promotes autoimmunity via TLR and JAK/STAT signaling

**DOI:** 10.1172/jci.insight.196144

**Published:** 2025-12-22

**Authors:** Bridget N. Alexander, Soojin Kim, Kristen L. Wells, Maya J. Hunter, Kevin P. Toole, Scott M. Wemlinger, Daniel P. Regan, Andrew Getahun, Mia J. Smith

**Affiliations:** 1 Barbara Davis Center for Diabetes,; 2 Department of Immunology and Microbiology, and; 3 Department of Pediatrics, University of Colorado School of Medicine, Aurora, Colorado, USA.; 4 Department of Microbiology, Immunology, and Pathology, Colorado State University, Fort Collins, Colorado, USA.

**Keywords:** Autoimmunity, Immunology, Autoimmune diseases, B cells

## Abstract

Autoimmunity arises when self-reactive B and T cells target the body’s own tissues, with B cells contributing through antigen presentation as well as production of autoantibodies and proinflammatory cytokines. Genome wide association studies (GWAS) and recent identification of loss-of-function gene variants in individuals with young-onset autoimmunity have highlighted a role for protein tyrosine phosphatase nonreceptor type 2 (PTPN2) in development of autoimmunity. While prior studies have focused on the mechanism of *Ptpn2* in T cells and other cell types, its function in B cells has not been explored. To test the B cell–intrinsic roles of *Ptpn2*, we generated a B cell*–*specific deletion of *Ptpn2* in mice (Mb1-Cre;*Ptpn2^fl/fl^*). We found that loss of *Ptpn2* in B cells promoted organ inflammation, increased the frequency of age/autoimmune-associated B cells (ABCs) and plasmablasts in the periphery, and increased circulating autoantibodies. Moreover, we found that *Ptpn2* acted as a negative regulator of the JAK/STAT and TLR7 pathways in B cells. In line with this, treatment of B cells from Mb1-Cre;Ptpn2^fl/fl^ mice with IFN-γ and TLR7 agonist lead to enhanced differentiation into ABCs. These findings highlight the critical roles of *Ptpn2* in B cell function and its potential as a key regulator in preventing B cell associated autoimmunity.

## Abstract 2

Loss of Ptpn2 in B cells disrupts immune tolerance and proper signaling, promoting inflammation and autoantibody production, which contributes to development of autoimmunity.

## Graphical abstract

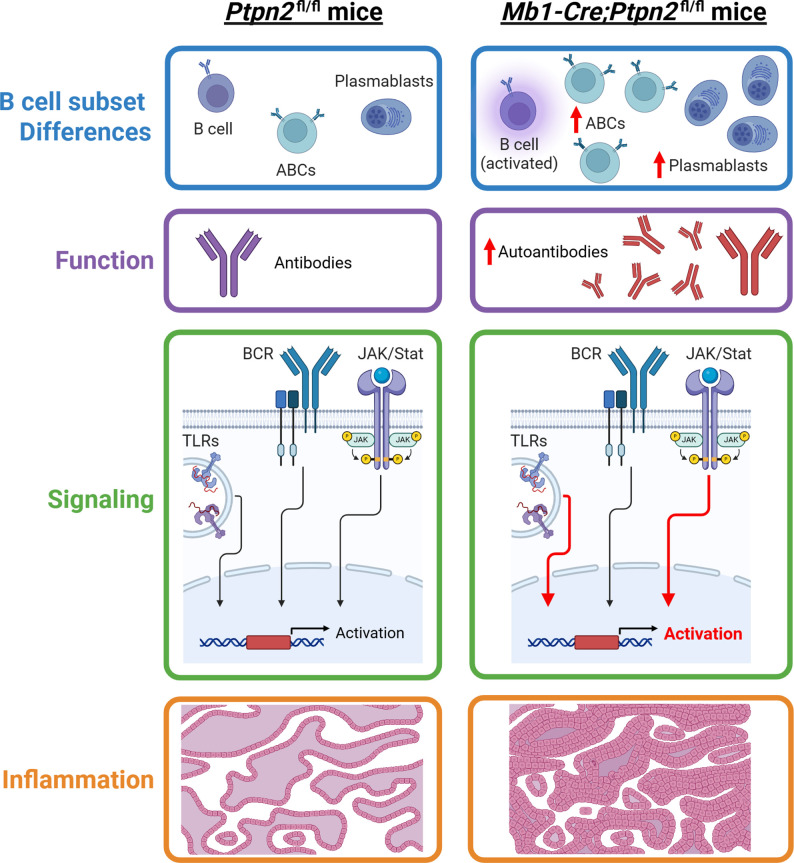

## Introduction

Autoimmunity arises when self-reactive B and T cells begin targeting the body’s own cells and tissues. B cells are thought to contribute to development of autoimmunity through 3 main effector functions: (a) autoantibody production, (b) (self)antigen presentation to autoreactive T cells, and (c) production of proinflammatory cytokines. While environmental factors contribute to the majority of autoimmune conditions, such as systemic lupus erythematosus (SLE), rheumatoid arthritis (RA), and type 1 diabetes (T1D), genetic predisposition remains the primary determinant of risk (1, 2). GWAS of various autoimmune disorders have implicated a wide array of polymorphisms in genes outside the HLA loci that are expressed in immune cells (3–5).

Noncoding SNPs in the protein tyrosine phosphatase nonreceptor type 2 (PTPN2; also known as T cell protein tyrosine phosphatase [TC-PTP]) are implicated in the pathogenesis of autoimmune diseases, including RA, Crohn’s disease, and T1D (6–10). While it is not entirely clear how these SNPs affect *Ptpn2* mRNA and protein levels, a previous study has shown that homozygosity for the PTPN2 SNP (rs1893217) results in 33%–50% reduction in *PTPN2* mRNA levels in human CD4^+^ T cells (11). In addition to common noncoding variants identified through GWAS, rare monogenic variants in *Ptpn2* have also been linked to early-onset autoimmunity and autoinflammatory disease. Case studies and exome sequencing efforts have identified heterozygous loss-of-function mutations in PTPN2 in pediatric patients with severe immune dysregulation, including early-onset Evan’s syndrome, SLE, and systemic inflammation (12). These individuals often exhibit heightened IFN responses and enhanced JAK/STAT signaling. Functional assays of patient-derived cells confirmed impaired *Ptpn2* activity, leading to excessive STAT1 and STAT3 phosphorylation and elevated proinflammatory cytokine production. These rare but functionally disruptive mutations underscore the critical role of *Ptpn2* in maintaining immune homeostasis and preventing aberrant lymphocyte activation in both the innate and adaptive immune compartments.

*Ptpn2* is expressed in various cell types, including both B and T cells. Initial studies detailing inducible deletion of *Ptpn2* in the hematopoietic compartment of adult mice demonstrated widespread inflammation and overt autoimmunity through enhanced T and B cell responses (13). T cells were skewed toward Tfh cells, while B cell development, activation, and production of anti-nuclear autoantibodies were enhanced, suggesting *Ptpn2* deletion compromises mechanisms that silence autoreactive lymphocytes (13). Specific deletion of *Ptpn2* in T cells (Lck-Cre; *Ptpn2*^fl/fl^ C57BL/6) has shown that *Ptpn2* has a range of functions in T cells, including inhibition of TCR signaling through dephosphorylation of LCK and FYN following antigen receptor stimulation (14), negative regulation of JAK/STAT signaling (15), as well as ability to influence T cell differentiation (14). Mice in which T cells lack *Ptpn2* develop widespread inflammation and autoimmunity that can occur in a nonautoimmune background strain, such as C57BL/6 (14). When crossed to the NOD mouse model, which develops spontaneous diabetes, deletion of *Ptpn2* in T cells leads to accelerated rates of diabetes with a heightened susceptibility to other autoimmune conditions, such as colitis and Sjögren’s syndrome (16). Moreover, when *Ptpn2* is deleted specifically in pancreatic β cells, mice demonstrate higher susceptibility to inflammatory stress through dysfunctional metabolic fitness (17). Taken together, these studies highlight a role for *Ptpn2* as an important negative regulator of T cell signaling and as a suppressor of inflammatory stress, which likely helps to prevent development of autoimmunity. However, given the important roles of B cells in development of autoimmunity, such as antibody production and autoantigen-presentation to T cells, knowledge of how *Ptpn2* impacts B cell activation may inform our understanding of development of autoimmunity.

In a healthy individual, autoreactive B cells are tolerized by 1 of 3 mechanisms: receptor editing, clonal deletion, or anergy. While receptor editing and clonal deletion occur centrally in the bone marrow, B cell anergy occurs in the periphery. Anergic B cells are characterized as being in a state of unresponsiveness that is mediated, in part, by upregulation of inhibitory signaling molecules, leading to an inability to differentiate into antibody secreting cells (18–20). Studies in mice indicate that the induction of anergy in autoreactive B cells is crucial to maintaining immunological tolerance and preventing autoimmune diseases (21–24). Previously, we analyzed the frequency of anergic B cells in the peripheral blood of subjects with varying autoimmune conditions compared with age/sex-matched controls. We found that individuals with T1D, autoimmune thyroid disease (AITD), and SLE have a significant decrease in the frequency of anergic B cells in their peripheral blood compared with control donors (25–28), suggesting that loss of B cell anergy likely contributes to development of autoimmunity in humans. Moreover, to understand the possible contribution of genetic risk alleles to loss of B cell anergy, we genotyped nonautoimmune first-degree relatives with varying levels of anergic B cells in their peripheral blood for T1D-associated genetic risk alleles. We found that individuals who carried the PTPN2 SNP (rs1893217) also had the lowest frequency of anergic B cells in their peripheral blood, suggesting that *Ptpn2* may play a role in controlling B cell tolerance (29).

Although *Ptpn2* is best characterized as a negative regulator of cytokine signaling through the JAK/STAT pathway, there is emerging evidence that it may also modulate TLR signaling, including TLR7 and TLR9. In myeloid cells, loss of Ptpn2 has been shown to enhance inflammatory responses to TLR ligands, including TLR7 and TLR8, through increased production of type I IFN and proinflammatory cytokines (30). While direct evidence for *Ptpn2* activity downstream of TLR7/9 in B cells is limited, these pathways are key mediators of innate immune activation in autoreactive B cells and contribute to autoimmune pathogenesis in diseases, such as SLE. Given that TLR7 and TLR9 activation in B cells leads to cytokine production, plasmablast differentiation, and class switching — processes that are also regulated by JAK/STAT signaling (31–37) — it is plausible that *Ptpn2* may act as a central modulator of these converging pathways. Nevertheless, its specific role in restraining TLR-driven signaling in B cells remains to be fully elucidated.

To better understand the B cell intrinsic role of *Ptpn2* in maintenance of B cell tolerance and prevention of autoimmunity, we generated B cell specific *Ptpn2*-KO mice using the Mb1-Cre allele (henceforth known as Mb1-Cre;*Ptpn2*^fl/fl^). We demonstrate that *Ptpn2* is an important regulator of B cell function and differentiation. B cell–specific loss of *Ptpn2* promotes organ inflammation, increases the frequency of age/autoimmune-associated B cells (ABCs) and plasmablasts, leads to production of autoantibodies, and acts as negative regulator of the JAK/STAT and TLR7 signaling pathways. Our results highlight the importance of *Ptpn2* in controlling activation of autoreactive B cells that can lead to the development of autoimmunity.

## Results

### Mb1-Cre;Ptpn2^fl/fl^ mice display increased immune cell infiltration into various organs.

**Figure 1:**
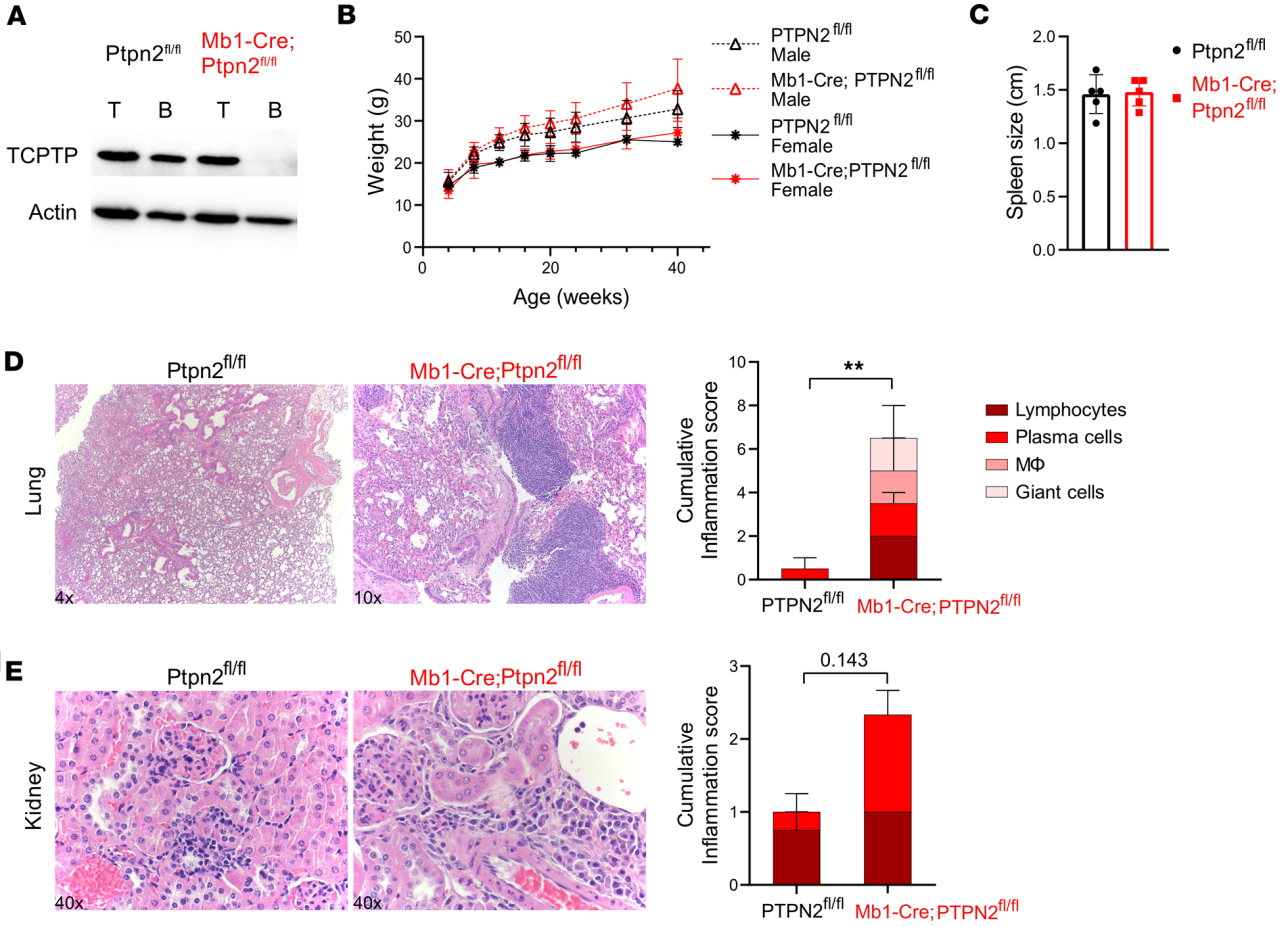
Elevated immune pathology in Mb1-Cre;*Ptpn2*^fl/fl^ mice. (**A**) Western blots confirming *Ptpn2* (TCPTP) deletion in B cells, not T cells; β-actin used as loading control. (**B**) One-year longitudinal body weight analysis in both sexes (*n* = 12–22). (**C**) Spleen sizes at 24 weeks (*n* = 5). (**D** and **E**) Representative histology of lung (4×) and kidney (40×) showing immune cell infiltrates in Mb1-Cre;*Ptpn2*^fl/fl^ mice with quantified cumulative inflammation score (*n* = 5). Data are shown as mean ± SEM, and **C**–**E** are representative of ≥ 3 independent experiments. **P < 0.01, Student’s *t* test.

Although previous research has linked *Ptpn2*-deficient T cells and hematopoietic stem cells to increased systemic inflammation and autoimmunity (13, 14), the exact role of *Ptpn2* in B cells is less well understood. To address this, we generated mice that lack *Ptpn2* expression in B cells on a C57BL/6 background by crossing a B cell–restricted Cre recombinase expressing mouse (Mb1-Cre) with *Ptpn2*^fl/fl^ mice. B cell–restricted *Ptpn2* deficiency was confirmed by Western blot (Figure 1A). To ensure sufficient time for potential differences to emerge, Mb1-Cre^+^ and Mb1-Cre^–^ littermates were allowed to age for more than 1 year. Throughout this period, body weight was monitored regularly, and no differences in weight were observed between Mb1-Cre;*Ptpn2*^fl/fl^ and control *Ptpn2*^fl/fl^ mice of either sex (Figure 1B). In addition, analysis of urinary protein to assess kidney function revealed no significant changes or differences between strains (data not shown), and there were no overt signs of autoimmunity in Mb1-Cre;*Ptpn2*^fl/fl^ mice compared with controls. Interestingly, while spleen size remained consistent across strains (Figure 1C), histological analysis in > 52-week-old *Ptpn2*-deficient mice revealed increased immune cell infiltrates in the lung and kidney (Figure 1, D and E, respectively) and, to a lesser extent, in the liver and pancreas (Supplemental Figure 1A; supplemental material available online with this article; https://doi.org/10.1172/jci.insight.196144DS1) compared with control mice. Specifically, the number of lymphocytes was markedly increased in the lungs and trending in the kidneys in *Ptpn2*-deficient mice (Figure 1, D and E, respectively). Histological analysis of 24- to 26-week-old mice revealed no significant immune infiltrates in the various organs of either strain (Supplemental Figure 1B). Taken together, B cell–specific *Ptpn2* deficiency did not lead to overt signs of disease or autoimmunity, but immune cell infiltration was increased in Mb1-Cre;*Ptpn2*^fl/fl^ mice ≥ 1 year old when compared with *Ptpn2-*sufficient controls.

### Mb1-Cre;Ptpn2^fl/fl^ mice have increased levels of serum Igs and autoantibodies.

**Figure 2:**
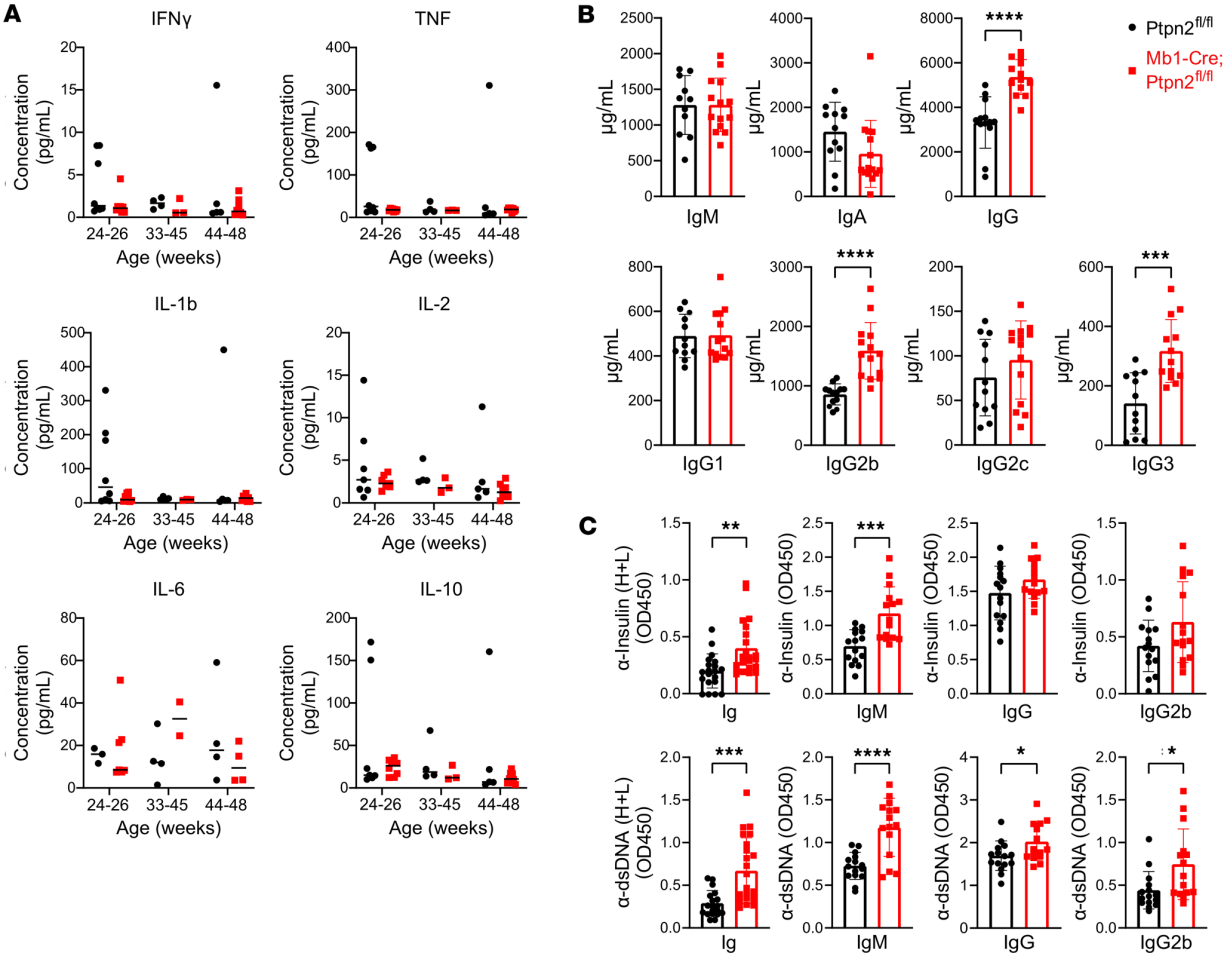
Increased (auto)antibodies in Mb1-Cre;*Ptpn2*^fl/fl^ mice. (**A**) MSD assay measured serum proinflammatory cytokine concentrations (pg/mL) (IFN-γ, TNF, IL-1β, IL-2, IL-6, IL-10) from mice aged 24–26 weeks (*n* = 8), 33–45 weeks (*n* = 3–4), or 44–48 weeks (*n* = 5–8). (**B**) ELISAs showing serum concentrations (μg/mL) of IgM, IgA, IgG, IgG1, IgG2b, IgG2c, and IgG3 in 24-week-old mice (*n* = 11–14). (**C**) Anti-insulin and anti-DNA autoantibodies measured by ELISAs, including total Ig, IgM, IgG, and IgG2b levels were determined (*n* = 15–23). Data are shown as mean ± SEM. *P < 0.05,**P < 0.01, ***P < 0.001, ****P < 0.0001, 2-tailed Student’s *t* test.

To better understand the observed inflammation, we next compared serum cytokine levels from the 2 strains at various ages. As shown in Figure 2A, across all ages and strains, there were no significant differences in the levels of IFN-γ, TNF, IL-1β, IL-2, IL-6, or IL-10 found in the serum. These findings demonstrate that B cell–specific loss of *Ptpn2* does not lead to altered levels of systemic cytokines. Given that the hematopoietic *Ptpn2*-KO mouse had increased serum Igs and autoantibodies (13), we next wanted to assess whether differences in circulating (auto)antibodies were also found in our mice. We observed an increase in total serum IgG levels, but not IgM or IgA, in the Mb1-Cre;*Ptpn2*^fl/fl^ mice compared with controls at 24 weeks of age (Figure 2B). To determine which IgG subclasses were responsible for the overall increase in serum IgG found in our B cell–specific *Ptpn2*-deficient mice, we analyzed IgG1, IgG2b, IgG2c, and IgG3 titers from the same mice. We found that IgG2b and IgG3 were significantly increased in Mb1-Cre;*Ptpn2*^fl/fl^ mice compared with controls (Figure 2B). Both IgG2b and IgG3 have been shown to be involved in early stages of inflammation and are associated with autoimmune disease progression (38–44).

Next, we determined whether Mb1-Cre;*Ptpn2*^fl/fl^ mice had an increase in serum autoantibodies compared with control mice. We found that Mb1-Cre;*Ptpn2*^fl/fl^ mice had a significant increase in anti-insulin and anti-dsDNA antibodies compared with controls (Figure 2C). Interestingly, while total serum IgM levels were similar between the 2 strains, we found that autoantigen-specific IgM was significantly increased for both anti-insulin and anti-dsDNA in the Mb1-Cre;*Ptpn2*^fl/fl^ mice compared with controls (Figure 2C). In addition, anti-dsDNA IgG and, specifically, IgG2b were significantly increased in the *Ptpn2* B cell–deficient mice. Anti-insulin IgG and IgG2b were increased but did not reach statistical significance in Mb1-Cre;*Ptpn2*^fl/fl^ mice compared with controls (Figure 2C). The other anti-insulin and anti-dsDNA Ig isotypes were not significantly different between the 2 strains (Supplemental Figure 2). Overall, these findings demonstrate that loss of *Ptpn2* in B cells leads to enhanced (auto)antibody production.

### Ptpn2 B cell–deficient mice display an increased frequency of immature B cells, loss of anergic (T3) B cells, and an increase in ABCs.

Given that the *Mb1* gene encodes CD79a and is expressed early during B cell development, we first wanted to determine if B cell development was altered in the bone marrow of Mb1-Cre;*Ptpn2*^fl/fl^ mice. While there was a slight increase in the frequency of small Pre-B cells in the Mb1-Cre;*Ptpn2*^fl/fl^ mice compared with controls, overall, the major developing B cell subsets in the bone marrow, as analyzed by Hardy fractions (45), were similar between the 2 strains (Supplemental Figure 3A). Central tolerance mechanisms such as receptor editing and clonal deletion are operative during B cell development. Since we observed increased autoantibody production in Mb1-Cre;*Ptpn2*^fl/fl^ mice, we wanted to determine if this was a consequence of a defect in central B cell tolerance. The ratio of kappa/lambda BCR light chain usage can be used as a surrogate for receptor editing (central tolerance) (46). We observed no significant difference in the kappa/lambda ratio (Supplemental Figure 3B) between the 2 genotypes, suggesting that the observed loss of B cell tolerance is likely peripheral.

**Figure 3:**
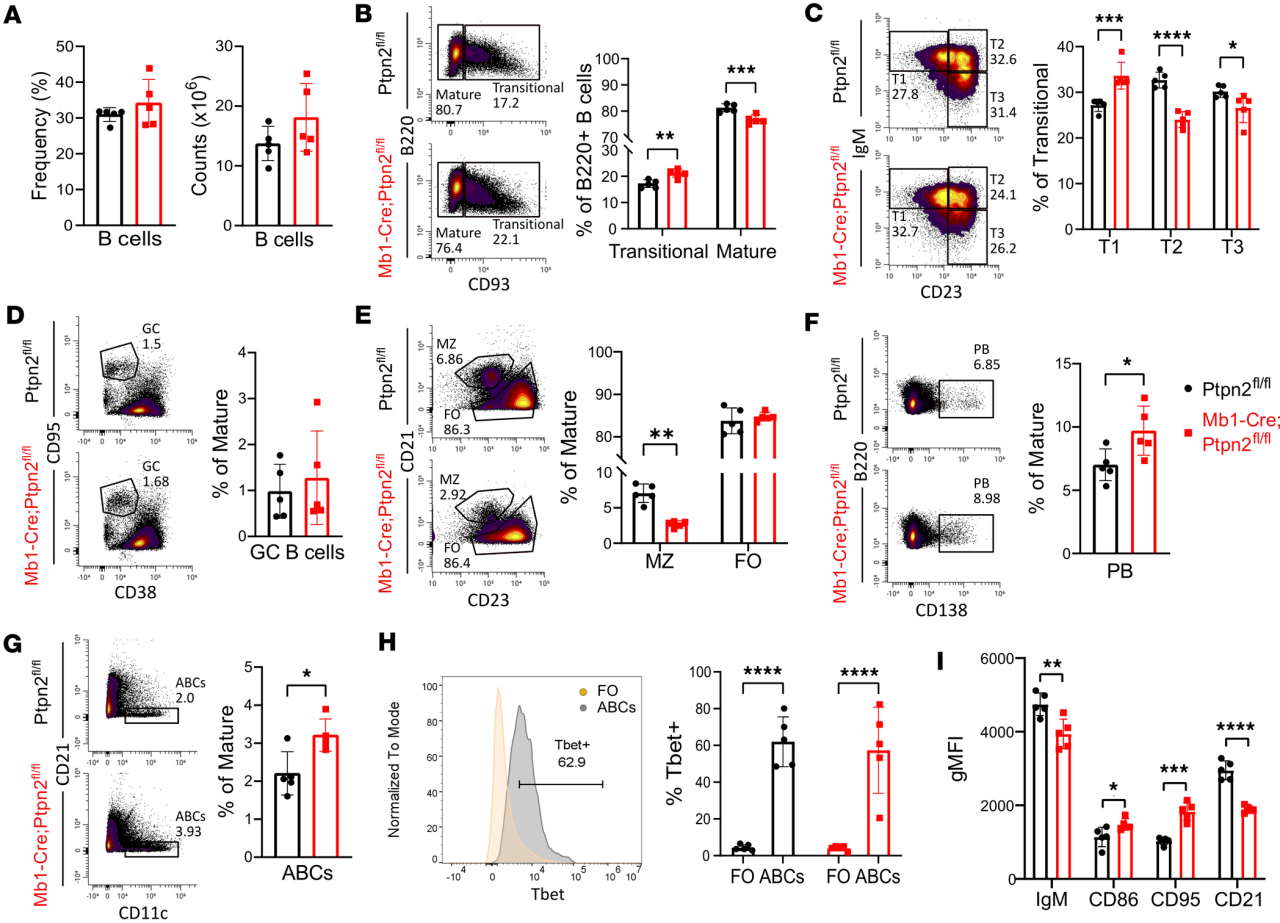
Increased B cell activation and expansion of autoreactive subsets in Mb1-Cre;Ptpn2^fl/fl^ mice. (**A**) In 24-week-old mice, total B cell number and frequency in spleens of Ptpn2^fl/fl^ and Mb1-Cre;Ptpn2^fl/fl^ mice. (**B**) Gating strategy and quantification of transitional (B220^+^CD93^+^) vs mature (B220^+^CD93^–^) B cells. (**C**) Subset analysis of transitional B cells: T1 (IgM^hi^CD23^lo^), T2 (IgM^hi^CD23^hi^), and T3 (IgM^lo^CD23^hi^), gated on B220^+^CD93^+^ cells. (**D**) Germinal center (GC; CD95^hi^CD38^lo^) B cells within mature B cells. (**E**) Follicular (FO; CD21^lo^CD23^+^) and marginal zone (MZ; CD21^hi^CD23^lo^) B cells within mature B cells. (**F** and **G**) Plasmablasts (PB; CD138^+^) and age-associated B cells (ABCs; CD21^–^CD11c^+^) gated from mature B cells. (**H**) T-bet expression (% T-bet^+^) in FO and ABC populations. (**I**) Geometric mean fluorescence intensity (gMFI) of activation markers (IgM, CD86, CD95, CD21) on mature B cells. Data are shown as mean ± SEM, representative of ≥ 3 independent experiments (*n* = 5). *P < 0.05, **P < 0.01, ***P < 0.001, ****P < 0.0001, 2-way ANOVA (**B**, **C**, **E**, and **H**) or Student’s *t* test (**A**, **D**, **F**, **G**, and **I**).

Next, we analyzed the absolute number and frequency of various B cell subsets found in the spleen of Mb1-Cre;*Ptpn2*^fl/fl^ mice compared with *Ptpn2^fl/fl^* controls (Supplemental Figure 4). We found no observable differences in B cell subsets between Mb1-Cre;*Ptpn2^wt/wt^* and *Ptpn2^fl/fl^* mice (Supplemental Figure 5), suggesting that neither the insertion of Cre in the CD79A locus nor the insertion of loxp sequences in the *Ptpn2* locus affect B cell development and differentiation. To minimize the effects of background genetics, we used Cre^–^ *Ptpn2*^fl/fl^ littermates as controls for Mb1-Cre;*Ptpn2*^fl/fl^ mice. Although we saw trending differences in young mice (8–10 weeks of age) (Supplemental Figure 6), we found more significant differences starting around 24 weeks of age. Consistent with our previous findings that spleen size was similar between the 2 strains, we found no difference in the absolute cell count or frequency of B220^+^ B cells in the spleen of our mice (Figure 3A and Supplemental Figure 7). When we compared the frequency of transitional (CD93^+^) B cells to mature (CD93^–^) B cells, we found that our Mb1-Cre;*Ptpn2*^fl/fl^ mice had a significant increase in the frequency and absolute number of transitional B cells with a respective decrease in the frequency, but not number, of mature B cells compared with control mice (Figure 3B and Supplemental Figure 7).

Transitional B cells can be further divided into T1, T2, and T3 subsets. T1 cells represent newly emigrated immature B cells from the bone marrow, while T2 cells represent an intermediate stage of transitional B cells that are not yet fully responsive to antigen stimulation. Nonautoreactive T2 cells will then mature further to become mature naive B cells in the periphery; however, if T2 cells bind to self-antigen, then they will enter the T3 compartment. Previous studies have demonstrated that the T3 compartment is enriched in autoreactive anergic B cells, which exhibit impaired responsiveness to antigen stimulation and fail to undergo proper activation and differentiation into antibody-secreting cells (ASCs) (47, 48). We found that Mb1-Cre;*Ptpn2*^fl/fl^ mice exhibit a significant increase in the frequency and trending increase in absolute number of T1 cells with a correlated decrease in both the T2 and T3 populations compared with control mice (Figure 3C and Supplemental Figure 7). These findings suggest that loss of *Ptpn2* in B cells may accelerate differentiation of transitional B cells through their various stages, ultimately leading to the reduction in the tolerized autoreactive T3 B cell population.

Next, we characterized the splenic mature B cell subsets in our 2 strains. First, we assessed germinal center (GC) B cells, which play a critical role in the selection of high-affinity plasma cells during the immune response (49–51) but also can give rise to autoantibodies, if dysregulated (52). While we observed increases in select Ig isotypes, as well as anti-insulin and anti-DNA antibodies in the serum of B cell specific *Ptpn2*-deficient mice (Figure 2, B and C), we did not observe any difference in the frequency or absolute number of GC B cells between the control and Mb1-Cre;*Ptpn2*^fl/fl^ mice (Figure 3D and Supplemental Figure 7). In addition, we found that Mb1-Cre;*Ptpn2*^fl/fl^ mice have a significant decrease in the frequency and absolute number of marginal zone (MZ) B compared with controls (Figure 3E and Supplemental Figure 7). MZ B cells have been shown to be enriched in autoreactivity and can rapidly differentiate into antibody secreting plasma cells (53). In line with this, we found Mb1-Cre;*Ptpn2*^fl/fl^ mice have a significant increase in the frequency and absolute number of plasmablasts (PBs) compared with the control mice (Figure 3F and Supplemental Figure 7), suggesting that differentiated MZ B cells may contribute to the observed increase in PBs.

We then determined whether our B cell specific Ptpn2-deficient mice exhibited differences in the frequency of age/autoimmune ABCs. ABCs have been shown to be enriched in autoreactivity, are increased in autoimmune prone mouse strains, as well as individuals with autoimmunity, and are precursors to PBs (54, 55). We found a significant increase in frequency and absolute number of ABCs (B220^+^CD21^lo^CD11c^+^) in our Mb1-Cre;*Ptpn2*^fl/fl^ mice compared with controls (Figure 3G and Supplemental Figure 7). To validate that these cells were truly ABCs, we assessed their intracellular expression of the transcription factor, T-bet, which is a classic marker of ABCs. As shown in Figure 3H, CD11c^+^CD21^lo^ B cells express high levels of T-bet, with a significantly higher percentage of T-bet^+^ cells compared with follicular (FO) B cells, confirming their identity as ABCs. Finally, we analyzed the activation status of B cells from Mb1-Cre;*Ptpn2*^fl/fl^ mice and found that they express markers consistent with recent activation. B cells lacking *Ptpn2* displayed decreased surface expression of IgM and CD21 and increased expression of CD86 and CD95 compared with control B cells, which may reflect increased activation (56–58) (Figure 3I). Taken together, loss of *Ptpn2* in B cells leads to enhanced production of (auto)antibodies, loss of anergic T3 and MZ B cells, an increase in ABCs and PBs, and changes in the surface expression of markers that are associated with B cell activation.

### T cells from Mb1-Cre;Ptpn2^fl/fl^ mice are skewed toward more effector/memory T cells.

**Figure 4:**
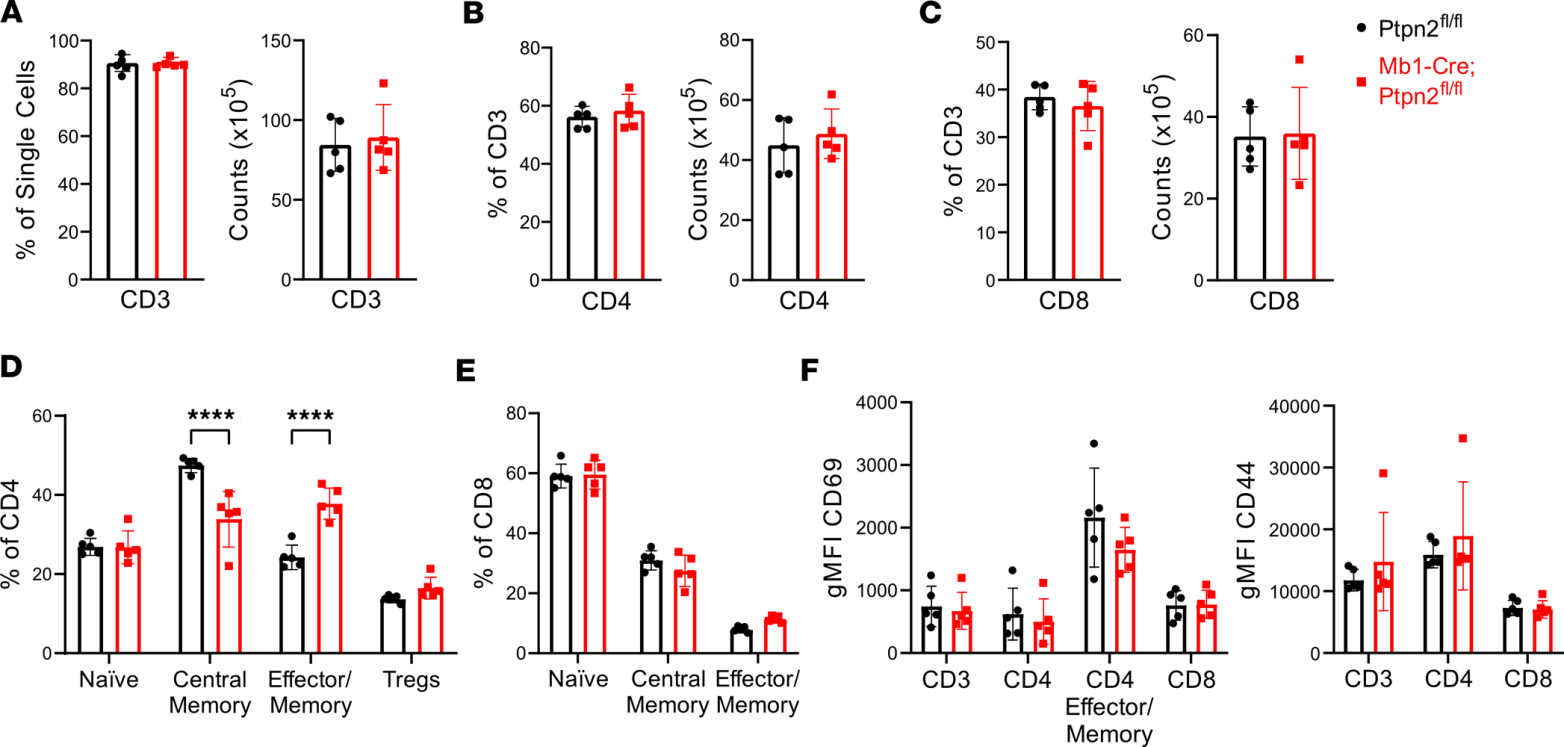
Mb1-Cre;*Ptpn2*^fl/fl^ mice have elevated CD4 effector/memory cells with no change in activation. (**A**–**C**) Number and frequency of splenic CD3 (**A**), CD4 (**B**), and CD8 (**C**) T cells in 24-week-old mice. (**D** and **E**) T cell subset distributions within CD4^+^ (**D**) CD8^+^ (**E**) populations: naive (CD3^+^, CD4^+^, CD62L^+^, CD44^lo^), central memory (CD3^+^, CD4^+^, CD62L^+^, CD44^+^), effector/memory (CD3^+^ CD4^+^, CD62L^lo^, CD44^+^), and Tregs (CD3^+^, CD4^+^, CD25^+^, CD127^+^, for CD4^+^ only). (**F**) CD69 and CD44 expression on CD3^+^, CD4, CD4 effector/memory (CD69^+^ only), and CD8 T cells. Data are shown as mean ± SEM and are representative of ≥ 3 independent experiments (*n* = 5, ****P < 0.0001, 2-way ANOVA).

Given that B cells are important contributors to the activation of T cells, we wanted to determine whether T cells would be affected by a B cell–specific *Ptpn2* deficiency. Similar to findings from the B cells, there was no change in the absolute cell count or percentage of CD3^+^ (Figure 4A), CD4^+^ (Figure 4B), or CD8^+^ T cells (Figure 4C) between the 2 strains. When we further characterized the CD4^+^ T cell subsets in the spleen, we found a significant decrease in the frequency of central memory T cells and a corresponding increase in CD4^+^ effector/memory T cells (Figure 4D). We found no significant differences in the CD8^+^ T cell subsets (Figure 4E). To determine whether T cells exhibit a more activated phenotype, we measured surface expression of CD69 and CD44 on T cells and found no difference in their expression between the 2 strains (Figure 4F). Based on these findings, loss of *Ptpn2* in B cells may aid the differentiation of CD4^+^ effector/memory T cells, but these cells do not appear more activated.

### Altered gene expression associated with JAK/STAT, TLR signaling, and ABCs in Ptpn2-deficient B cells.

**Figure 5:**
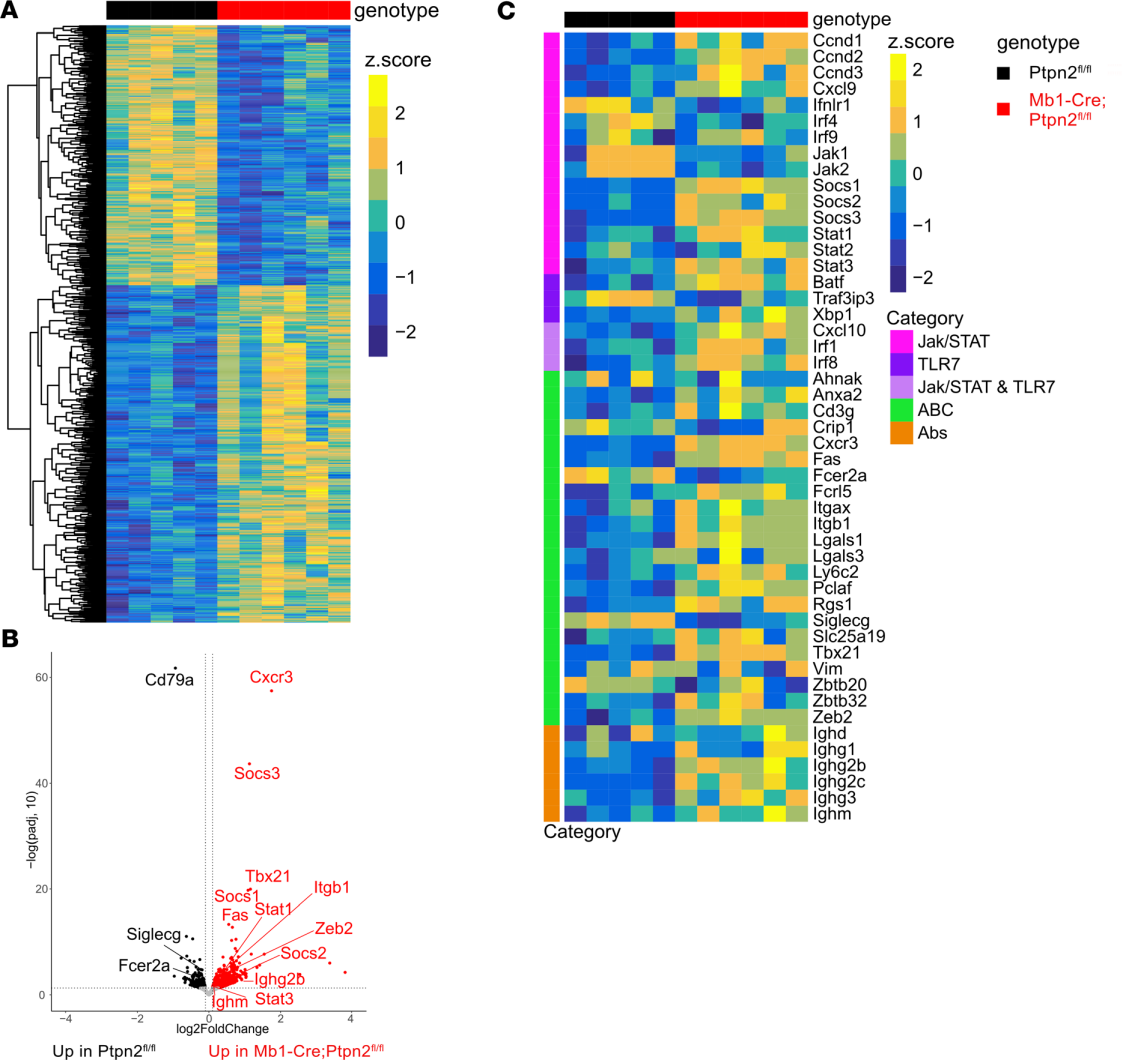
Bulk RNA-Seq reveals enhanced expression JAK/STAT and ABCs-associated genes in *Ptpn2*-deficient B cells. (**A**) Heatmap of all differentially expressed genes (DEGs) (*n* = 5–6). (**B**) Volcano plot of DEGs. Red dots indicate increased in Mb1-Cre;*Ptpn2*^fl/fl^ mice; black dots indicate increased in the controls. Labeled genes are hand-selected (log_2_FC > 0.5). (**C**) Heatmap of selected genes involved in JAK/STAT and TLR signaling, ABC development, and Ig isotypes.

To gain an unbiased view of the phenotypic differences of B cells with and without *Ptpn2*, we conducted bulk RNA-Seq analysis on B cells isolated from Mb1-Cre;*Ptpn2*^fl/fl^ mice and controls. Principal component analysis (PCA) demonstrated clear separation between the 2 genotypes, underscoring distinct transcriptional profiles (Supplemental Figure 8). RNA expression analysis revealed 970 differentially expressed genes (DEGs), with 548 being upregulated in the Mb1-Cre;*Ptpn2*^fl/fl^ mice (Figure 5A and Supplemental Table 1). A full list of bulk RNA-Seq DEGs are provided in Supplemental Table 1. To further investigate the variable gene expression between the strains, we performed gene set enrichment analysis. We found that the Mb1-Cre;*Ptpn2*^fl/fl^ mice exhibit a significant increase in expression of genes associated with JAK/STAT signaling, such as Socs1, Socs2, Socs3, Stat1, and Stat3, consistent with previous reports that *Ptpn2* acts as a negative regulator of the JAK/STAT signaling pathway (Figure 5, B and C) (15, 59).

In addition to JAK/STAT-related changes, we observed upregulation of genes involved in TLR signaling, particularly TLR7 and TLR9 pathways, including *Batf*, Cxcl10, *Xbp1*, *Irf1*, and *Irf8,* as well as a decrease in *Traf3ip3*, a negative regulator of TLR7 signaling (Figure 5, B and C). These genes are associated with enhanced innate immune activation, B cell differentiation, and IFN responses, suggesting that the loss of *Ptpn2* may potentiate TLR-driven signaling cascades. Notably, we did not observe differential expression of core proximal TLR7/9 signaling components such as *MyD88*, *IRAK1*, *IRAK4*, *TRAF6*, or *IRF7*, which may reflect their constitutive expression, posttranscriptional regulation, or the fact that Ptpn2 regulates further downstream. Moreover, *Ptpn2*-deficient B cells had altered expression of genes associated with the ABC subset, including Cxcr3 (up), Tbx21 (Tbet) (up), Fas (up), Itgb1 (up), Zeb2 (up), Siglecg (down), and Fcer2a (down) (Figure 5, B and C), consistent with our B cell phenotyping analysis (60).

When analyzing Ig isotype expression from the 2 strains, we found that B cells from Mb1-Cre;*Ptpn2*^fl/fl^ mice had an overall increase in IgG subclasses IgG2b, IgG2c, and IgG3. This finding is consistent with the serum titers, indicating an increase in class-switching (Figure 5, B and C). However, we also noted modest increases in IgM and IgG1 transcript levels in the *Ptpn2*-deficient group despite no corresponding change in total IgM or IgG1 Ig levels, as measured by ELISA. This apparent discrepancy likely reflects that transcript levels in bulk RNA-Seq do not always correlate directly with secreted protein abundance, particularly for Igs, which can persist in serum over time. These differences underscore the complexity of linking transcript abundance to functional protein output, particularly for Igs.

### B cell receptor (BCR) signaling is normal in Ptpn2-deficient B cells.

Given that others reported that a T cell–specific KO of *Ptpn2* results in an increase in TCR signaling (14, 61–64), we wanted to assess whether loss of *Ptpn2* in B cells alters BCR signaling in our Mb1-Cre;*Ptpn2*^fl/fl^ mice. After stimulation with F(ab’)_2_ anti-IgM, we measured the phosphorylation of BCR signaling proteins Src, CD79a, Syk, PLCγ2, and AKT. We found no significant difference in the level of phosphorylation of these proteins between B cells from Mb1-Cre;*Ptpn2*^fl/fl^ and control mice (Supplemental Figure 9A). Similarly, when we measured BCR-induced intracellular calcium mobilization, no significant difference between B cells from Mb1-Cre;*Ptpn2*^fl/fl^ and control mice was observed (Supplemental Figure 9B). Unlike studies from the T cell–specific deletion of *Ptpn2*, we found no effect of *Ptpn2* deletion on BCR signaling.

### Hyperresponsiveness of the JAK/STAT pathway in Mb1-Cre;Ptpn2^fl/fl^ mice.

**Figure 6:**
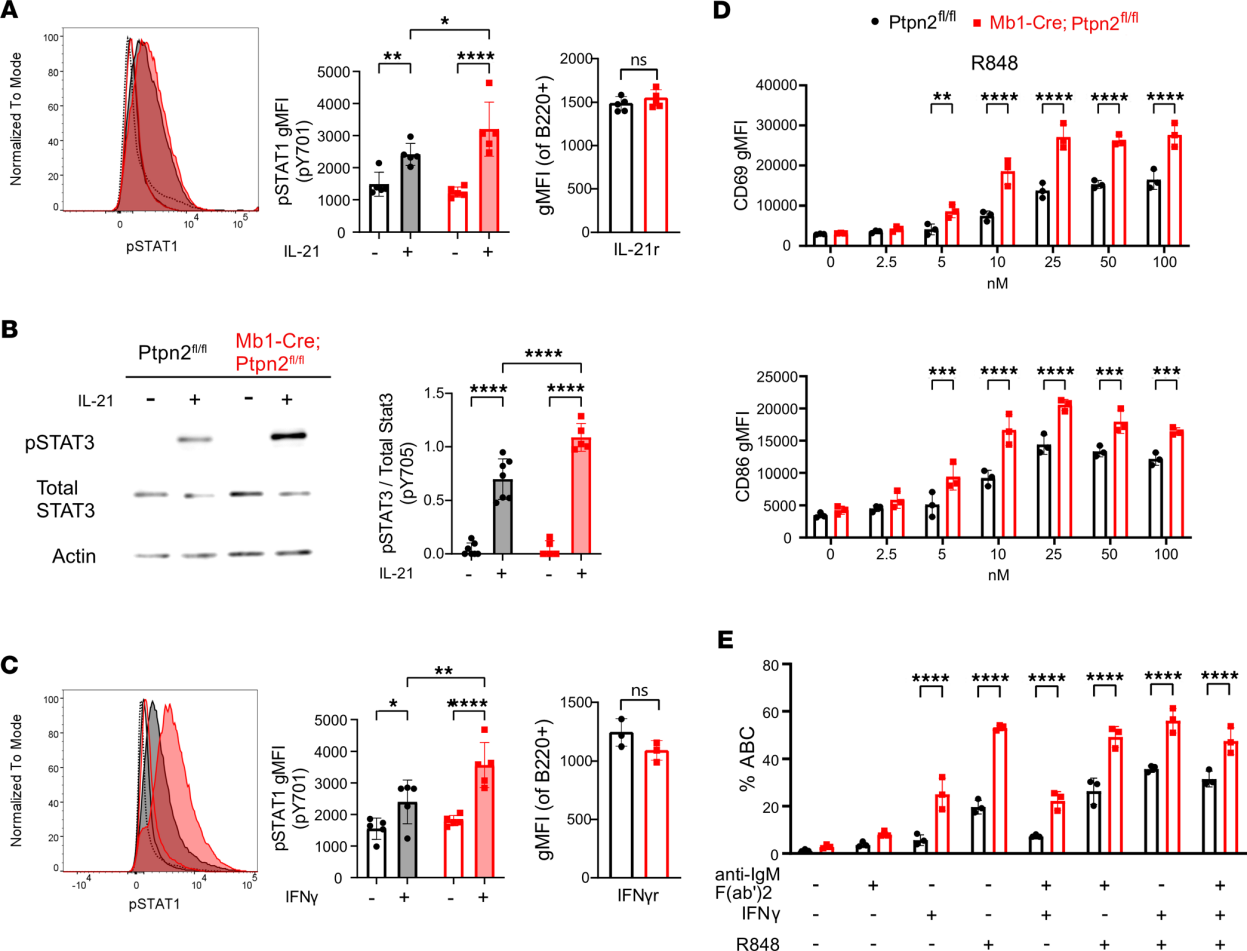
B cell–specific *Ptpn2* deficiency increases JAK/STAT and TLR7 signaling and ABC differentiation. (**A**) In 24-week-old mice phosphorylation of STAT1 pY701 after IL-21 stimulation, with IL-21 receptor expression. Data shown are gated on B220^+^ cells (*n* = 5). (**B**) Western blot and quantification of STAT3 phosphorylation (pY705) after IL-21 stimulation. pSTAT3 pY705 level was normalized to total STAT3 (*n* = 5–7). (**C**) Phosphorylation of STAT1 pY701 after IFN-γ stimulation, with IFN-γ receptor expression. Data shown are gated on B220^+^ cells (*n* = 5). (**D**) CD69 and CD86 expression after R848 (TLR7) stimulation at indicated doses (*n* = 3). (**E**) ABC frequency (B220^+^, CD93^–^, CD21^–^, CD11c^+^) after 48 hours in vitro stimulation (*n* = 3). All experiments utilized 24-week-old mice. Data are shown as mean ± SEM and are representative of ≥ 3 independent experiments. *P < 0.05, **P < 0.01, ***P < 0.001, ****P < 0.0001, 2-way ANOVA.

Based on the results from our bulk RNA-Seq and previous studies demonstrating *Ptpn2* acts as a negative regulator of the JAK/STAT signaling pathway (15, 59), we analyzed JAK/STAT signaling in B cells from our KO mice. Splenic B cells were stimulated with either recombinant mouse IL-21 or IFN-γ. IL-21 is a potent STAT activator and plays a key role in B cell differentiation into plasma cells in the GC (31, 65). After IL-21 stimulation, phosphorylation of Stat1 (Figure 6A) and Stat3 (Figure 6B) were found to be increased in *Ptpn2*-deficient B cells compared with controls. Importantly, no difference in surface expression of IL-21 receptor was observed (Figure 6A). We next sought to confirm JAK/STAT hyperresponsiveness by utilizing an alternative cytokine. IFN-γ has previously been shown to facilitate B cell differentiation into ABCs, promoting activation, class switching, and survival of autoreactive B cells (66, 67). Similar to our findings using IL-21, we observed elevated phosphorylation of Stat1 in Mb1-Cre;*Ptpn2*^fl/fl^ mice after stimulation with IFN-γ, with both strains showing equal surface expression of IFN-γ receptors (Figure 6C).

### Mb1-Cre;Ptpn2^fl/fl^ mice are hyperresponsiveness to TLR7 stimulation.

Because certain TLRs have been associated with autoimmunity, we wanted to determine whether *Ptpn2* affects signaling through TLR7 and TLR9. TLR7 has been implicated in various autoimmune diseases, such as SLE, as its activation can trigger the production of autoantibodies and contribute to disease pathology (68). Similarly, TLR9 contributes to anti-DNA autoantibody responses (69), although its contributions to disease pathology are more context dependent (70–73). Using the B cell activation markers CD69 and CD86 as a readout for stimulation, we performed dose-response curves for splenic B cells stimulated with the TLR agonists R848 (TLR7) and ODN1668 (TLR9). After TLR7 stimulation with R848, Mb1-Cre;*Ptpn2*^fl/fl^ B cells displayed significantly increased levels of both activation markers beginning at 5 nM R848 to our highest dose of 100 nM (Figure 6D). Similarly, we found that *Ptpn2*-deficient B cells are more responsive to TLR9 stimulation, albeit only at higher ODN1668 concentrations (Supplemental Figure 10). Taken together, these results indicate that loss of *Ptpn2* in B cells leads to hyperresponsiveness of TLR7 and, to a lesser extent, TLR9.

To assess whether JAK/STAT and TLR7 hyperresponsiveness was present in younger mice, we repeated cytokine and TLR7 stimulations in B cells from 6- to 8-week-old mice. While both IL-21 and IFN-γ induced robust phosphorylation of Stat1, no significant differences were observed between *Ptpn2*-deficient and control B cells (Supplemental Figure 11A). In addition, hyperresponsiveness to TLR7 stimulation was not observed in *Ptpn2*-deficient mice compared with controls in 6- to 8-week-old mice (Supplemental Figure 11B). These data indicate that the hyperresponsiveness observed in our 24-week-old mice develops over time, which is consistent with our findings of alterations in frequencies of B cell subsets, such as ABCs and PBs, tissue infiltration, and autoantibody levels that were found in older mice but not young mice.

### B cells from Mb1-Cre;Ptpn2^fl/fl^ mice more readily differentiate into ABCs in vitro.

Since TLR7 and IFN-γ are known to be key players in the differentiation of ABCs (67, 74–76) and loss of *Ptpn2* in B cells leads to hyperresponsiveness to TLR7 and IFN-γ, we wondered whether B cells deficient in *Ptpn2* may be predisposed to differentiate into ABCs in vitro. To test this, we isolated splenic B cells from 24-week-old mice and depleted preexisting ABCs to ensure any observed increase in the frequency of ABCs was due to new generation and not proliferation of preexisting ABCs. We then stimulated CD11c^–^ B cells with different combinations of R848, IFN-γ, and F(ab’)_2_ anti-IgM, which are known to promote ABC differentiation (76). As shown in Figure 6E, we found that any combination containing IFN-γ or R848 led to a significant increase in the frequency of newly generated ABCs when B cells lack *Ptpn2* expression. These findings suggest that B cell–intrinsic loss of *Ptpn2* leads to hyperresponsiveness to TLR7 and JAK/STAT stimulation, which together enhances the generation of ABCs.

## Discussion

In this study, we report the first direct evidence to our knowledge of a critical B cell intrinsic role for *Ptpn2* in controlling B cell responsiveness, activation, and loss of B cell tolerance. Mice deficient in *Ptpn2* only in their B cells exhibit immune infiltration into the lung and kidney and have increased (auto)antibodies in their serum; furthermore, their B cells express increased markers of activation and are skewed toward more autoreactive and pathogenic B cell subsets, including PBs and ABCs. While it has previously been found that *Ptpn2* negatively regulates the TCR signaling pathway and JAK/STAT signaling in T cells (13, 14), our data show that the absence of *Ptpn2* in B cells results in the hyperresponsiveness to TLR7 stimulation and cytokine stimulation through the JAK/STAT pathway, with no evidence of an effect on the BCR signaling pathway. These findings highlight differential roles of *Ptpn2* in B cells versus T cells and how not all negative regulators function similarly in lymphocytes.

The hyperresponsiveness of *Ptpn2*-deficient B cells to TLR7 stimulation is of particular interest, given the important role TLR7 plays in promoting loss of B cell tolerance (68, 71, 77). Previous findings in B cells have demonstrated that the TLR7 and JAK/STAT pathways converge in the following way. Stimulation through TLR7 leads to production of cytokines, such as type I IFN, that then stimulate the B cell in an autocrine fashion, resulting in stimulation of the JAK/STAT pathway, leading to activation and differentiation of B cells (37). In line with this possibility, when B cells are stimulated through TLR7 in the presence of the JAK inhibitor, tofacitinib, overall activation, as evidenced by CD69 and CD86 expression, is reduced (Supplemental Figure 12). Hence, loss of *Ptpn2*, a negative regulator of the JAK/STAT pathway, leads to an observed hyperresponsiveness of both the JAK/STAT and TLR7 pathways.

Interestingly, the majority of the B cell phenotypes observed in our *Ptpn2*-deficient mice, including changes in B cell subset frequencies and absolute cell counts, autoantibody levels, and signaling hyperresponsiveness, occurred in older (24-week-old) mice but not in younger (6- to 8-week-old) mice, even though *Ptpn2* was deleted early during B cell development. It is possible that age-related immune dysfunction, accumulated inflammation, and defective immune tolerance mechanisms lead to an altered peripheral B cell repertoire, as evidenced by increased ABCs and PBs, which is accelerated by the loss of *Ptpn2* in B cells. Future studies are needed to address these possibilities.

While our findings emphasize a B cell–intrinsic role for *Ptpn2* in restraining ABC formation and autoantibody production, *Ptpn2* expression in other cell types, particularly T cells, is also critical for preventing autoimmunity. In NOD mice with T cell–specific deletion of *Ptpn2*, diabetes onset and incidence — often accompanied by other autoimmune comorbidities — were accelerated (16). Notably, this T cell–specific deficiency also led to increased CD4^+^ Th differentiation and Tfh cell polarization with a corresponding increase in B cells in pancreatic islets, as well as increased GC B cells and anti-insulin autoantibodies (16). These findings suggest that *Ptpn2* deficiency in T cells alone can drive robust B cell responses, autoantibody production, and autoimmunity, albeit on an autoimmune background, highlighting the complex interplay between T and B cells in disease pathogenesis. Similarly, hematopoietic-specific *Ptpn2* deletion via Poly I:C administration induces an exacerbated autoimmune phenotype, including system inflammation, elevated inflammatory cytokines, increased Tfh and GC B cells, and enhanced B cell proliferation ex vivo (13). While the phenotype of our mice is milder, we found a similar increase in anti-nuclear antibodies and Igs, along with enhanced IL-21–induced STAT3 signaling and modest tissue inflammation. T cell–restricted *Ptpn2* deficiency on a nonautoimmune prone background also results in modest tissue inflammation as mice age and the generation of autoantibodies (13). These findings suggest that the severe phenotype in hematopoietic KOs arises from synergistic dysregulation of both T and B cells, combined with type I IFN response induced by Poly I:C. Importantly our study demonstrates that *Ptpn2* deletion in B cells alone is sufficient to drive spontaneous inflammation and autoantibody production, even on a nonautoimmune background, through dysregulation of TLR7 and JAK/STAT pathways. Thus, while T cell–intrinsic *Ptpn2* deficiency drives more pronounced systemic autoimmunity, our results demonstrate that B cell–intrinsic *Ptpn2* loss alone contributes to development of autoreactivity, highlighting the independent and complementary roles of *Ptpn2* in these lymphocyte compartments.

Our results have implications for human disease. While SNPs in *Ptpn2* have been associated with increased susceptibility to autoimmune conditions, identifying the functional consequences of these SNPs has been difficult, especially since these minor variants typically confer mild risk to disease. While *Ptpn2* is expressed ubiquitously throughout the body, most studies have focused on the role of *Ptpn2* in T cells in suppressing activation of autoreactive T cells. Here, we show that *Ptpn2* also plays an important role in controlling B cell tolerance to self-antigen, which increases our understanding of how these SNPs in *Ptpn2* may affect other cells of the immune system and lead to development of autoimmunity. In line with this, a recent study identified 6 heterozygous germline *PTPN2* mutations in patients who presented with pediatric onset autoimmunity (12). These variants resulted in frameshifts, nonsense mutations predicted to encode truncated proteins, or missense mutations. Importantly, peripheral blood cells from these patients displayed alterations in their T cells, characterized by hyperresponsiveness to cytokine stimulation through the JAK/STAT pathway, as well as differences in their B cells, with an increase in CD11c^+^ B cells (aka ABCs) present in their blood compared with control donors (12). These findings parallel observations in patients with STAT3 gain-of-function mutations, which also show increased ABCs and autoimmunity, suggesting a shared downstream mechanism linking PTPN2 loss and dysregulated STAT signaling (78). Hence, our findings that loss of *Ptpn2* in B cells leads to hyperresponsiveness through the TLR7 and JAK/STAT pathways, leading to an increase in ABCs and autoantibodies, may help explain the observed increase in ABCs in patients with PTPN2 mutations. Moreover, therapies targeting the JAK/STAT pathway, such as tofacitinib and baricitinib, have emerged as effective options for the treatment of autoimmune disorders, such as RA, SLE, and T1D (79–82). While the proposed mechanism(s) of action of JAK inhibitors do not typically focus directly on their role in B cells, our results suggest that these inhibitors could help control rogue activation of self-reactive B cells and/or compensate for the potential loss of function of *Ptpn2* in individuals who carry genetic risk alleles in *Ptpn2* by blocking the phosphorylation of STATs. Future studies of the direct effect of JAK inhibitors on B cells are needed to fully elucidate their effect on controlling loss of B cell tolerance.

In this study, we generated B cell–specific deletion of *Ptpn2* in mice on the C57BL/6 genetic background, which is resistant to developing autoimmunity. Nevertheless, our mice display evidence of development of autoimmunity. Genetic variants of PTPN2 associated with increased risk for autoimmune disease result in reduced *Ptpn2* expression levels (6, 12, 83–85). While the effects of haploinsufficiency of *Ptpn2* in B cells remain to be determined, as well as its ability to synergize with other genetic risk factors, our study highlights the crucial role of *Ptpn2* in regulating B cell signaling and immune tolerance. The loss of *Ptpn2* leads to heightened signaling activity, dysregulated B cell activation, and a shift toward autoreactivity, which may contribute to the development of autoimmune responses. Taken together our findings provide a look at the B cell intrinsic role of *Ptpn2*, as well as insights into the molecular mechanisms controlling B cell fate decisions.

## Methods

### Sex as a biological variable.

Data from both sexes were evaluated, and no sex-specific effects were observed. Results from both sexes were combined in all figures and statistical analyses.

### Mice.

Generation of *Ptpn2*^fl/fl^.C57BL/6J was previously described (86). We received the mice from Lori Sussel’s laboratory at the University of Colorado School of Medicine. Mb1-Cre.C57BL/6J [B6.C(Cg)-*Cd79atm1(Cre)Reth*/EhobJ, Jax# 020505] were purchased from Jackson Laboratory. Cross breeding between *Ptpn2*fl/fl.C57BL/6J and Mb1-Cre. C57BL/6J was set up to generate *Ptpn2*fl/fl.C57BL/6J or Mb1-Cre;*Ptpn2*fl/fl.C57BL/6J mice. No backcross was necessary since these modifying genes were already in C57BL/6J strain. Mice were housed at the animal facility at the University of Colorado School of Medicine and were fed a standard chow diet with free access to food and water under specific pathogen–free conditions. Unless otherwise indicated, 24- to 26-week-old male and female mice were used for all experiments. No differences were observed between males and females of either genotype. Aged-matched littermates were used in each experiment.

### Cell and tissue processing.

Spleens and bone marrow (from 1 femur and 1 tibia) were harvested in IMDM (Gibco, 12-440-053) supplemented with 5% FBS, 1 mM sodium pyruvate, 50 μg/mL gentamicin, 100 U/mL penicillin/streptomycin, and 2 mM l-glutamine. Single-cell suspensions were prepared by mechanical disruption. RBCs were lysed with 1 mL of RBC lysis buffer (BioLegend, 420302) for 1 minute at room temperature (RT). Cells were subsequently washed and resuspended in complete medium (IMDM + 5% FBS).

### Flow cytometry.

Single cell suspensions from splenocytes and/or bone marrow cells were incubated with optimized dilutions of anti-mouse antibodies for 15 minutes at 4°C in FACS buffer (1X PBS + 1% FBS). Cells were washed twice with FACS buffer before being fixed with 2% paraformaldehyde (PFA) in FACS.

For B cell phenotyping, cells were stained with B220-BV510 (BioLegend,103248); CD19-PeCy7 (BioLegend, 121120); IgM-BV711 (BioLegend, 406539); IgD-APC-Fire 750 (BioLegend, 405744); CD11c-PE (BioLegend, 117307); CD11c-PE-Dazzle 594 (BioLegend, 117347); CD138-BV785 (BioLegend, 142534); CD93-BUV737 (BD, 741800); CD23-BUV805 (BD, 741922); CD21-BV421 (BioLegend, 123421); CD3-FITC (BioLegend, 100204); IL-21r-APC (BioLegend, 131909); and IFN-γ r-APC (BioLegend, 113605). To stain for Tbet expression, the cells were fixed with 2% PFA following cell surface staining, pelleted, resuspended in ice-cold 100% methanol and left overnight at –80°C. Cells were washed once with FACS buffer, stained for 1 hour at RT with Tbet-PeCy7 (BioLegend, 644824) in FACS buffer, washed twice with FACS buffer, and then fixed with 2% PFA.

For activation markers, cells were stained with B220, CD69-BV421 (BioLegend, 104527); CD86-BV785 (BioLegend, 105043); CD95-APC-Fire 810 (BioLegend, 152624); Lambda-APC (BioLegend, 407306); and Kappa-FITC (Southern Biotech, 1170-02). For Hardy factions, cells were stained with antibodies against B220-AF647 (BioLegend, 103226); CD43-APC-Cy7 (BioLegend, 121219); BP-1-PE (BD, 553735); CD24-PeCy7 (BioLegend, 138507); IgM-BV605 (BioLegend, 406523); and IgD-BV711 (BioLegend, 405731). For T cell phenotypes, cells were stained with antibodies against, B220, CD3, CD4-BV785 (BioLegend, 100552); CD8-BV650 (BioLegend, 100742); CD44-AF647 (BioLegend, 103018); CD62L-PE (BioLegend, 104408); CD25-BV421(BioLegend, 102043); and CD127-PeCy7 (BioLegend, 121120). Viability stains included Zombie Green (BioLegend, 423112), Zombie NIR (BioLegend, 423105), or L/D Fixable Blue (Invitrogen, L34961). Single-color controls were used for compensation. Flow cytometry was performed on a Cytek Aurora spectral flow cytometer (Cytek Biosciences), and data analyzed with FlowJo software version 10.8.1or CellEngine (CellCarta).

Cell populations were defined as: transitional B cells (B220^+^, CD93^+^), T1 (B220^+^, CD93^+^, IgM^+^, CD23^lo^), T2 (B220^+^, CD93^+^, IgM^+^, CD23^hi^), T3 (B220^+^, CD93^+^, IgM^lo^, CD23^hi^), mature B cells (B220^+^, CD93^–^), GC B cells (B220^+^, CD93^–^, CD95^hi^, CD38^lo^), MZ B cells (B220^+^, CD93^–^, CD21^hi^, CD23^lo^), FO B cells (B220^+^, CD93^–^, CD21^lo^, CD23^+^), PBs (B220^+^, CD93^–^, CD138^+^), ABCs (B220^+^, CD93^–^, CD21^lo/–^, CD11c^+^, Tbet^+^), CD4 T cells (CD3^+^, B220^–^, CD4^+^), CD8 T cells (CD3^+^, B220^–^, CD8^+^), CD4 naive T cells (CD3^+^, B220^–^, CD4^+^, CD62L^+^, CD44^lo^), CD4 central memory T cells (CD3^+^, B220^–^, CD4^+^, CD62L^+^, CD44^+^), CD4 effector/memory T cells (CD3^+^, B220^–^, CD4^+^, CD62L^lo^, CD44^+^), CD4 Tregs (CD3^+^, B220^–^, CD4^+^, CD25^+^, CD127^+^), CD8 naive T cells (CD3^+^, B220^–^, CD8^+^, CD62L^+^, CD44^lo^), CD8 central memory T cells (CD3^+^, B220^–^, CD8^+^, CD62L^+^, CD44^+^), and CD8 effector/memory T cells (CD3^+^, B220^–^, CD8^+^, CD62L^lo^, CD44^+^). Gating strategy is shown in Supplemental Figure 4.

### TLR stimulation.

Splenic B cells were purified as a CD43^–^ fraction using anti-CD43 beads (Miltenyi Biotec, 130-049-801) and cultured at 4 × 10^6^ cells/mL for 18 hours at 37°C with TLR7 agonist R848 (InvivoGen, tlrl-r848-1), TLR9 agonist ODN1668 (InvivoGen, tlrl-1668), or JAK inhibitor tofacitinib (Cell Signaling Technologies, 14703s) at the indicated concentrations. Cells were pelleted, stained for 30 minutes at 4°C in FACS buffer, washed twice, and then resuspended in 2% PFA. B cells were analyzed for upregulation of the surface activation markers CD86 and CD69 by flow cytometry.

### In vitro ABC differentiation cultures.

Splenic B cells were purified as a CD43^–^ fraction using anti-CD43 beads and subsequently depleted of ABCs using anti-CD11c beads (Miltenyi Biotec, 130-125-835). ABC depleted B cells were cultured at 5 × 10^6^ cells per mL for 48 hours at 37°C at various conditions, as indicated. TLR7 agonist R848 was used at 1 μg/mL; (Fab′)2 anti-IgM (Jackson Laboratory, 115-006-075) was used at 5 μg/mL; and IFN-γ (BioLegend, 575304) at 100 U/mL. Cells were pelleted, stained for 15 minutes with antibodies recognizing B220, CD21, and CD11c at 4°C in FACS buffer, washed twice, resuspended in 2% PFA, pelleted, resuspended in ice cold 100% methanol, and left overnight at –80°C. Cells were washed once with FACS buffer, stained for 1 hour at RT with anti-Tbet in FACS buffer, washed twice with FACS buffer, and fixed with 2% PFA. Cells were then analyzed for the frequency of B cells that differentiated into ABCs of the total B cell population.

### Phosflow assays.

Splenocytes (1 × 10^7^ cells/mL) were rested for 30 minutes at 37°C in serum free IMDM (Gibco) before being stimulated with 10 μg/mL F(ab’)_2_ goat anti-mouse IgM (Jackson Laboratory, 115-006-075) for 3 minutes at 37°C or left unstimulated. Cells were immediately fixed with 2% PFA (wt/vol.) at 37°C, pelleted, and resuspended in Cytofix/Cytoperm (BD, 51-2090KZ) and placed on ice for 30 minutes. Cells were washed 3 times with Perm/Wash (BD, 554723), stained for 1 hour at 4°C with antibodies listed below in Perm/Wash, washed twice with Perm/Wash, and then resuspended in 2% PFA. Cells were stained with antibodies against B220; pAKT-AF647 (BD, 560343); pZap70/Syk-PeCy7 (BD, 561458); pPLCγ2-PE (BD, 558490); pCD79a-AF647 (Cell Signaling, 29742S); pSrc-PE (Invitrogen, 12-9034-42); or isotype controls: IgG1 Isotype control-PE (BioLegend, 400407); IgG1 Isotype control-AF647 (BioLegend, 400136); and IgG1 Isotype control-PeCy7 (BioLegend, 400125).

For pSTAT stimulation, splenocytes (1 × 10^7^ cells/mL) were rested for 1 hour at 37°C in serum free IMDM (Gibco) before being stimulated with either 50 ng/mL carrier-free rIL-21 (BioLegend, 574504) or 10 ng/mL carrier-free rIFN-γ for 10 minutes at 37°C or left unstimulated. Cells were immediately fixed with 2% PFA (wt/vol.) at 37°C for 5 minutes, pelleted, and resuspended in ice-cold 100% methanol and left overnight at –80°C. Cells were washed once with FACS buffer, stained for 1 hour at RT with the antibodies listed below in FACS buffer, washed 2 times with FACS buffer, and then resuspended in 2% PFA. Cells were stained with antibodies against B220, pSTAT1-PE (BD, 612564), pSTAT5-APC (BioLegend, 936906), or isotype controls.

### MSD assay.

Cytokine concentrations in mouse serum were measured using a Meso Scale Discovery (MSD) multiplex immunoassay, V-PLEX Proinflammatory Panel 1 (mouse) Kit (Meso Scale Diagnostics), following the manufacturer’s instructions. All samples were assayed in duplicate, and the average value was used for statistical analysis.

### Serum ELISA.

Microtiter plates were coated with 10 μg/mL goat anti-mouse Ig (Southern Biotech, 5300-05), 10 μg/mL recombinant human insulin (Sigma-Aldrich), or 10 μg/mL calf thymus DNA (Invitrogen) in PBS overnight at 4°C, followed by incubation with blocking buffer solution (2% BSA [wt/wt]) for 1 hour at RT. Sera from 24-week-old mice were serially diluted in blocking buffer and incubated in 96-well plates for 2 hours at RT. Between all steps, the plates were washed 4 times with PBS containing 0.05%Tween-20 (wt/vol.) (Sigma-Aldrich). Horseradish peroxidase–conjugated goat anti-mouse IgM, IgA, IgG, IgG1, IgG2b, IgG2c, or IgG3 (Southern Biotech, 5300-05) was added to the cells, followed by development with 3,3’,5,5’-tetramethylbenzidine (TMB) (Invitrogen, 002023), used to detect antibody binding. Reactions were stopped using 1M HCL (Sigma-Aldrich, H9892). Antibody binding was measured at an absorbance of 450 nm using a Biotek Synergy H1 Hybrid Reader (Aligent), and data were analyzed using Prism software version 10 (Graphpad Software). Ig concentrations were determined based on standards (Southern Biotech, 5300-01).

### Intracellular free calcium assay.

For measurements of intracellular free calcium concentration ([Ca^2+^]_i_), splenocytes (2 × 10^7^/mL in IMDM medium containing 2% FBS) were simultaneously stained with anti-B220-Alexa647 (BioLegend, RA3-6B2), Fab anti-IgM (Jackson ImmunoResearch, 115-007-020) conjugated to Dylight 488, as a nonstimulating measure of BCR expression, and loaded with 5 μM Indo-1 acetoxymethyl (Indo1-AM) (Molecular Probes) for 45 minutes at 37°C. After washing once with IMDM with 2% FBS, the cells were resuspended at 1 × 10^7^ cells/mL in IMDM with 2% FBS in a 500 μL volume. Prior to acquisition, cell suspensions were warmed to 37°C. Indo-1 was excited with a 355 nm laser, Ca^2+^-bound Indo-1 was detected using a 379/28 bandpass filter, and unbound Indo-1 was detected using a 450/50-410LP bandpass filter. Relative free calcium concentration was determined by the ratio bound/unbound Indo-1. After the baseline was established by analysis for 30 seconds, cells were stimulated with 10 μg/mL F(ab’)_2_ goat anti-mouse IgM (Jackson ImmunoResearch), and data were collected for an additional 2 minutes and 30 seconds. Relative mean [Ca^2+^]_i_ was measured using a BD LSRFortessa X-20 Cell Analyzer (BD Biosciences) and analyzed using FlowJo software (BD). The area under the curve was calculated in FlowJo. Events analyzed were B220^+^ and gated on equal IgM expression.

### Histology.

Lung, liver, kidney, pancreas, and tissue from 24- or 52-week-old mice were fixed in 10% neutral buffered formalin and then submitted for routine histological processing, embedding, sectioning, and staining with H&E. Histopathologic evaluation of lung, liver, kidney, and pancreas was performed in blinded fashion by a board-certified veterinary pathologist at the Comparative Pathology Laboratory at Flint Animal Cancer Center, Colorado State University. Organ slides were evaluated using an Olympus BX43 microscope and were assessed as normal or abnormal; if abnormal, they were semiquantitatively scored for inflammation, including cell type and degree, fibrosis, and necrosis, along with an accompanying qualitative histological description of morphological changes.

### Western blots.

Cell lysates were prepared by isolating splenic B (CD43^–^ fraction) and T cells (CD43^+^ fraction) using CD43 MACS beads (Miltenyi, 130-049-801) and LS columns (Miltenyi, 130-042-401) and lysing in NP40 lysis buffer (1% NP40, 150 mM NaCl, 50 mM Tris-HCl pH 7.4, 1 mM EDTA) supplemented with 100 μg/mL PMSF and Protease Phosphatase Inhibitor Cocktail (Cell Signaling Technology, 5872S). Equivalent of 5 × 10^5^ cells were loaded in each well. *PTPN2* was detected using primary anti-TCPTP (TC45) antibody (Cell Signaling Technology, 58935) and secondary HRP conjugated anti-rabbit IgG goat antibody (Cell Signaling Technology, 7074S).

For pSTAT3 analysis, isolated B cells (1 × 10^7^ cells/mL) were stimulated with 50 ng/mL rIL-21 carrier-free for 10 minutes at 37°C or left unstimulated, before being lysed with NP40 lysis buffer. The equivalent of 5 × 10^5^ cells was loaded in each well. Anti-pSTAT3 antibody (Cell Signaling Technology, 9145S) and secondary HRP conjugated anti-rabbit IgG goat antibody (Cell Signaling Technology, 7074S) were used to detect pSTAT3. Each membrane was stripped and reblotted with anti-STAT3 (Cell Signaling Technology, 9139S) and secondary HRP conjugated anti-mouse IgG goat antibody (SouthernBiotech, 1030-05) to detect total STAT3. HRP conjugated anti-β-actin (Santa Cruz Biotechnology, SC-47778) was used to detect β-actin as control for loading and normalization between samples. No significant difference in β-actin and total STAT3 was detected between samples. The proteins on the membrane were detected using SuperSignal West Pico PLUS Chemiluminescent Substrate (Thermo Scientific, PI34577) and imaged using Sapphire Biomolecular Imager (Azure Biosystems). Integrated density was measured using Image J (NIH).

### Bulk RNA-Seq.

Splenic B cells were isolated as a CD43^–^ fraction using anti-CD43 beads (Miltenyi Biotec, 130-049-801) and pellets frozen at –80°C until RNA extraction. The purity of this particular batch was not tested; however, the estimated purity is > 95% based on previous experience using this method. Total RNA was extracted using the RNeasy Plus Mini Kit (Qiagen, 74134). RNA quality was assessed using a Tecan Infinite M2 Pro for purity using the 260:280 ratio of 1.8:2.2 and at concentration of 200 ng total and Agilent Technologies Tape Station 4200 for integrity using a RIN score of 8–10. mRNA libraries were prepared using the TruSeq Stranded mRNA Library Prep Kit (Illumina) and sequenced on an Illumina NovaSeq X platform with 150bp paired-end reads.

Raw reads were trimmed using cutadapt (version 4.8, python version 3.10.14; https://doi.org/10.14806/ej.17.1.200) (87) and aligned to the mouse GRCm38 genome using STAR (version 2.7.11b) (88). Gene expression was quantified with featureCounts (version 2.0.6) (89), and differential expression analysis was conducted using DESeq2 (version 1.44.0) (90) with R version 4.4.1. Batch correction was performed using combatseq through the sva package (version 3.52.0) (91, 92). All plots were made using ggplot2 (version 3.5.1) in R.

### Statistics.

Data was analyzed using Prism software version 10 (Graphpad Software). An unpaired 2-tailed Student’s *t* test was used to compare differences between groups and a 2-way ANOVA was used to analyze data means with repeated measures when appropriate. *P* < 0.5 was considered significant.

### Study approval.

All experiments were performed in accordance with the regulations and with approval of the University of Colorado Denver IACUC.

### Data availability.

Values for all data points in graphs can be found in the Supporting Data Values file. Analysis can be replicated using code available on github, https://github.com/CUAnschutzBDC/smith_bridget_rnaseq (commitID: c1691fa8d4ac50874359350c7a3afbd06d763070); with docker images for preprocessing: https://hub.docker.com/r/kwellswrasman/rnaseq_general; and differential expression and plotting: https://hub.docker.com/r/kwellswrasman/rna_seq_r

All code is available at https://github.com/CUAnschutzBDC/smith_bridget_rnaseq

The raw and processed sequencing data generated in this study have been deposited in the Gene Expression Omnibus (GEO) database under accession code GSE295350.

## Author contributions

BNA, SK, AG, and MJS designed the experiments. BNA, SK, MJH, KPT, and SMW performed the experiments. BNA, SK, KLW, MJH, KPT, and DPR analyzed the data. BNA conducted and analyzed all signaling, phenotyping, and in vitro experiments. SK and KPT performed and analyzed all western blots. BNA and MJH ran and analyzed the ELISAs. SMW ran and BNA analyzed the calcium flux. KLW performed bioinformatic analysis and DPR performed histological analysis. BNA, AG, and MJS wrote the manuscript.

## Funding support

This work is the result of NIH funding, in whole or in part, and is subject to the NIH Public Access Policy. Through acceptance of this federal funding, the NIH has been given a right to make the work publicly available in PubMed Central.

- NIH grants K01OD028759 (MS), R03OD036470 (MS), R01AI84949 (MS)
- The Boettcher Foundation AWD-212445 (MS).
- The DRC P30DK116073 (LS).

## Supplementary Material

Supplemental data

Supplemental data set 1

Unedited blot and gel images

Supporting data values

## Version 1 12/22/2025

Electronic publication
