# Supplementary material for: Deletion of Ptpn2 in B cells promotes autoimmunity via TLR and JAK/STAT signaling
Source: JCI Insight. 2025 Dec 22;10(24):e196144. doi: 10.1172/jci.insight.196144 (PMC12890481; doi:10.1172/jci.insight.196144)
Supplement: Supplemental data [file jciinsight-10-196144-s095.pdf]

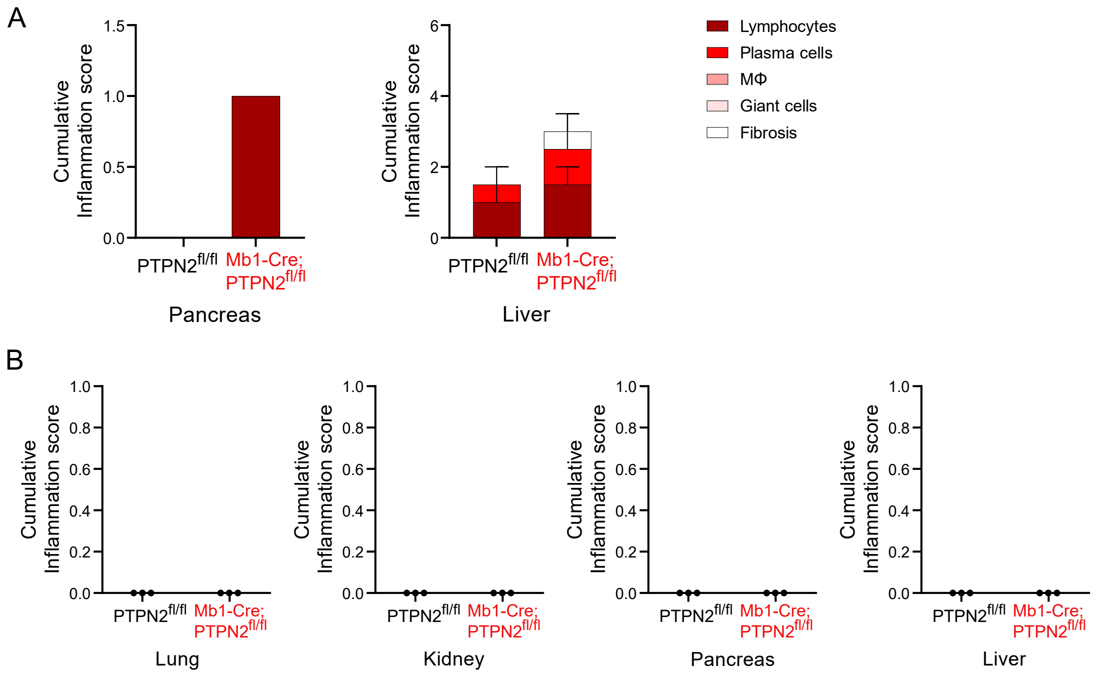

Supplemental Figure 1. Limited multi-organ inflammation in *Ptpn2*-deficient mice. A. Cumulative inflammation score of pancreas and liver from ≥1 year mice**. B.** Cumulative inflammation scores from 24 week old mice. Data is shown as mean ± SEM and is representative of ≥3 independent experiments. (n=3-5, Student's t test).

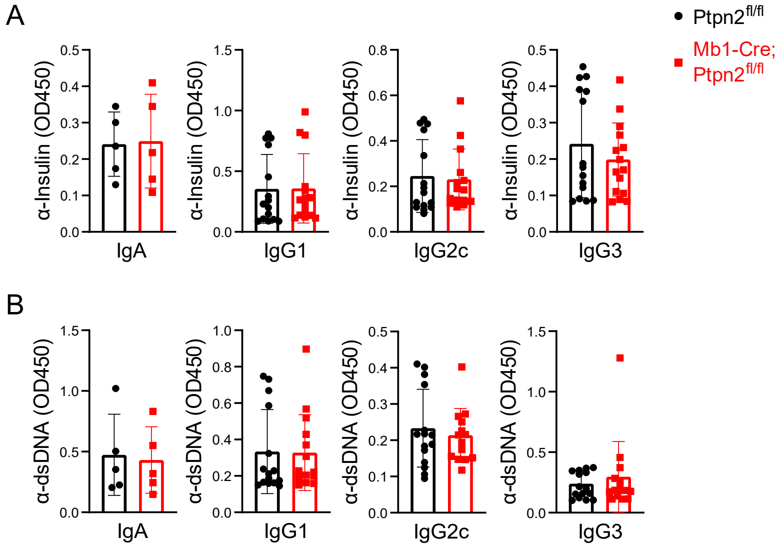

Supplemental Figure 2. Expanded isotype analysis of insulin and dsDNA autoantibodies. In 24 week old mice **A.** Anti-insulin and **B.** anti-DNA autoantibodies measured by ELISAs, including IgA, IgG1, IgG2c, and IgG3 levels were determined. Data is shown as mean ± SEM (n = 5-23, Student's t test).

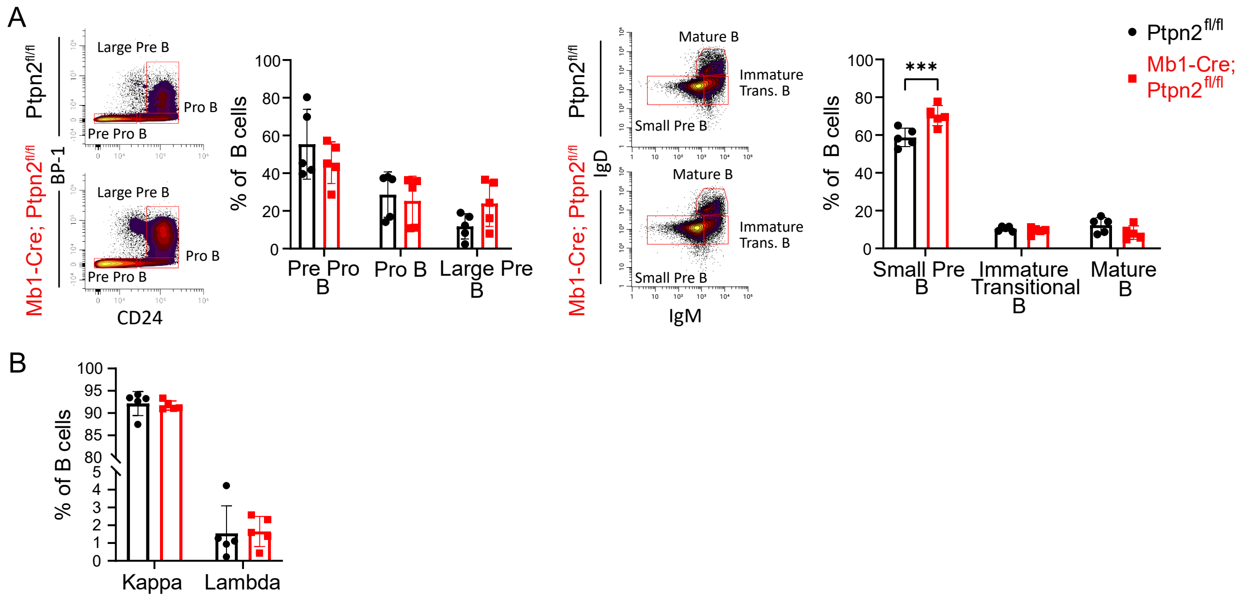

Supplemental Figure 3. B cell development. In 24 week old mice **A.** Gating strategy and quantification of Hardy Fractions (as percentage of B220+ B cells). **B.** Frequency of kappa and lambda (as percentage of B220+, CD19+ B cells). Data is shown as mean ± SEM and is representative of ≥3 independent experiments. (n=5, ***P < 0.001, 2-way ANOVA).

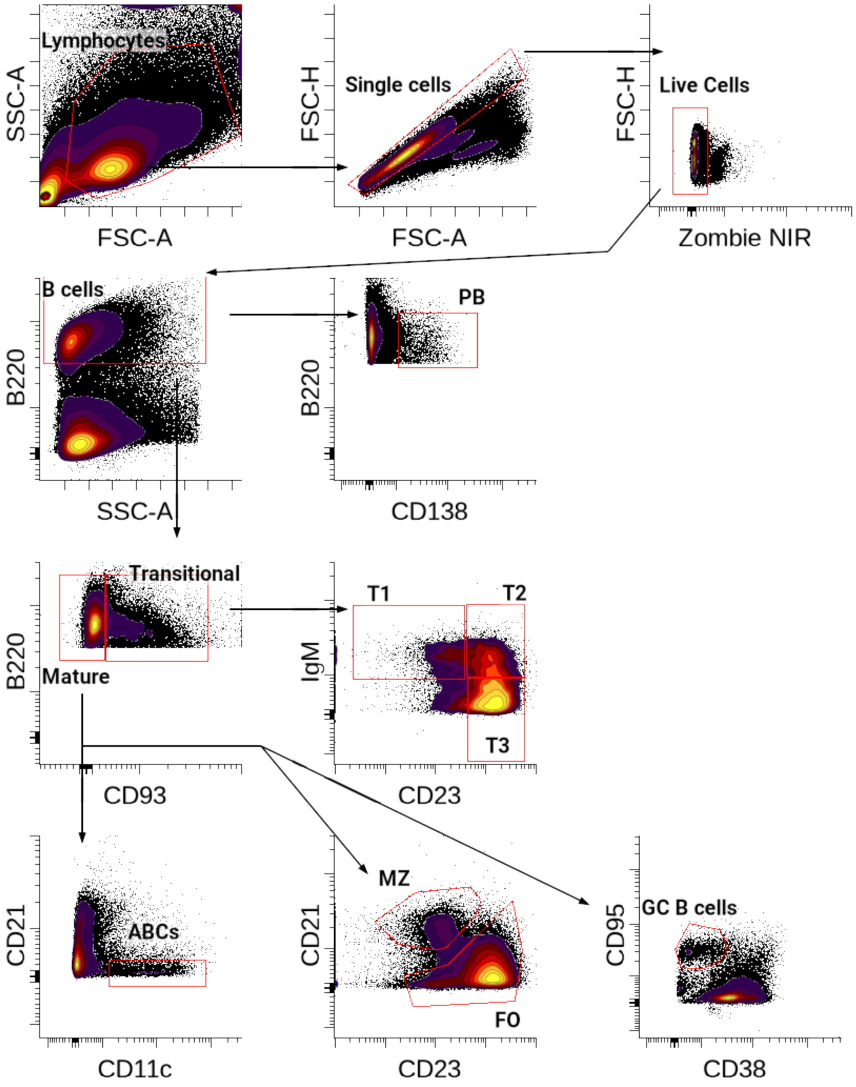

Supplemental Figure 4. Gating scheme. Gating strategy to differentiate B cell populations.

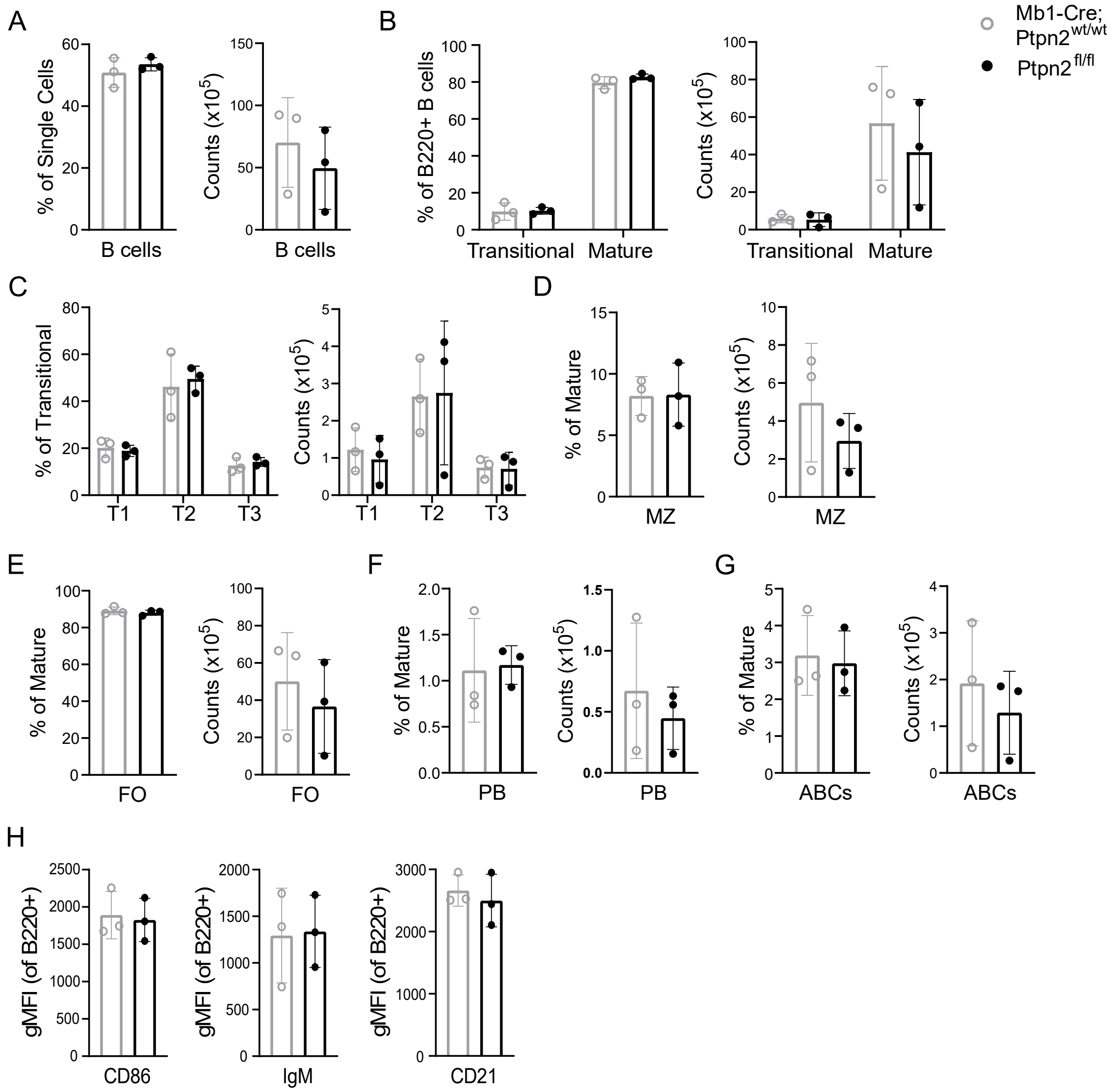

Supplemental Figure 5. Splenic B cell phenotypes are similar between Mb1-cre; *Ptpn2*$^{WT/WT}$ and *Ptpn2*$^{fl/fl}$ mice. In 24 week old mice, absolute cell counts and frequencies of **A.** total B cells, **B. t**ransitional and mature B cells, **C.** transitional subsets (T1, T2, T3), **D.** Marginal zone (MZ) B cells, **E.** Follicular (FO) B cells, **F.** Plasmablasts (PBs), and **G.** Age-associated B cells (ABCs). **H.** Geometric mean fluorescence intensity (gMFI) of activation markers (IgM, CD86, CD21) on mature B cells. Data are shown as mean ± SEM, representative of ≥3 independent experiments (n=3, 2-way ANOVA (B,C) or Student's t-test (A,D,E,F,G,H).

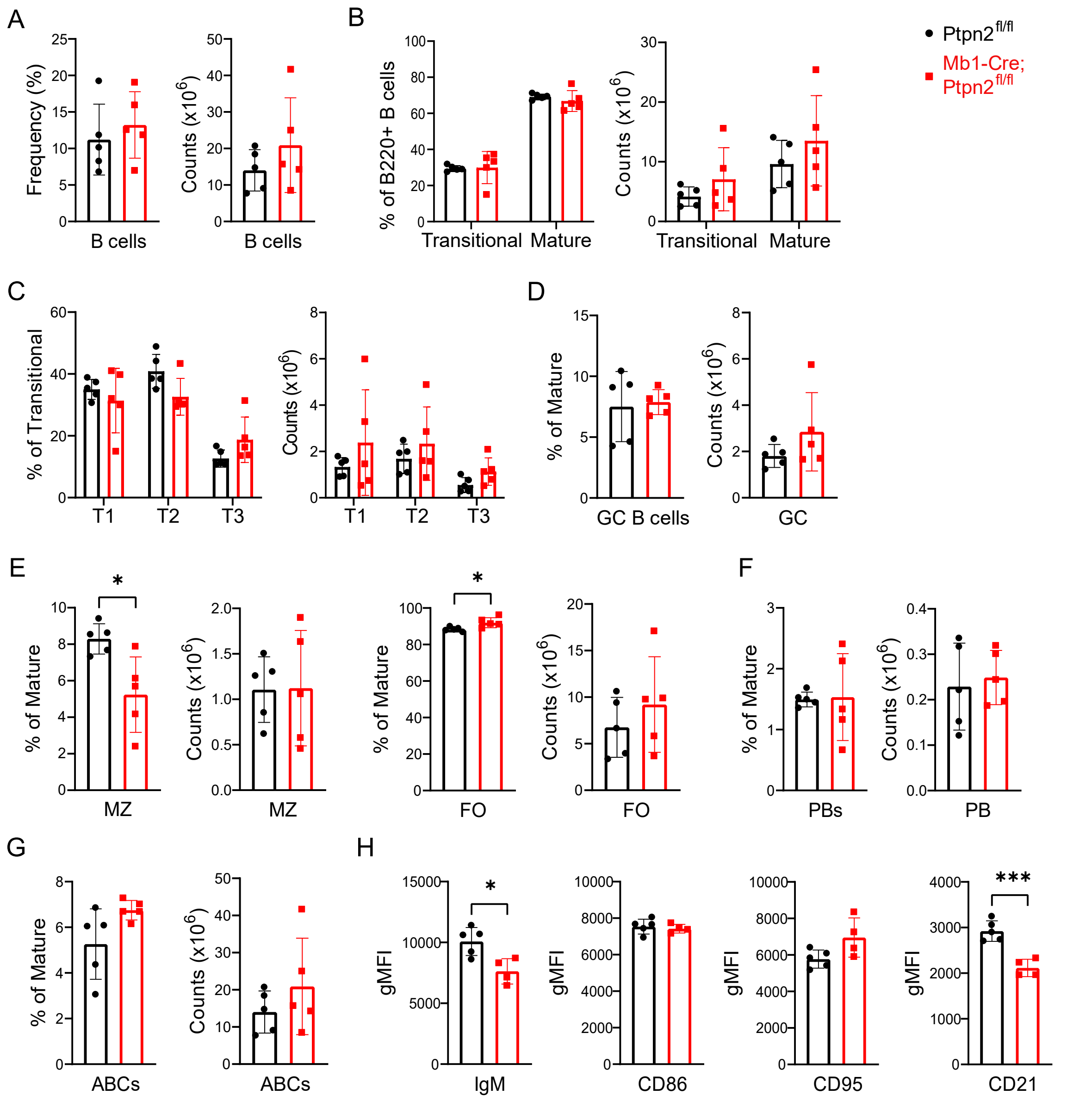

Supplemental Figure 6. B cell subsets in young (8-10 week old) mice. Absolute cell counts and frequencies of **A.** total B cells, **B.** Transitional and mature B cells, **C.** transitional subsets (T1, T2, T3), **D.** Germinal center (GC) B cells, **E.** Marginal zone (MZ) B cells and Follicular (FO) B cells, **F.** Plasmablasts (PBs), and **G.** Age-associated B cells (ABCs). **H.** Geometric mean fluorescence intensity (gMFI) of activation markers (IgM, CD86, CD95, CD21) on mature B cells. Data are shown as mean ± SEM, representative of ≥3 independent experiments (n=4-5, *P < 0.05, ***P < 0.001, 2-way ANOVA (B,C) or Student's t-test (A,D,E,F,G,H)).

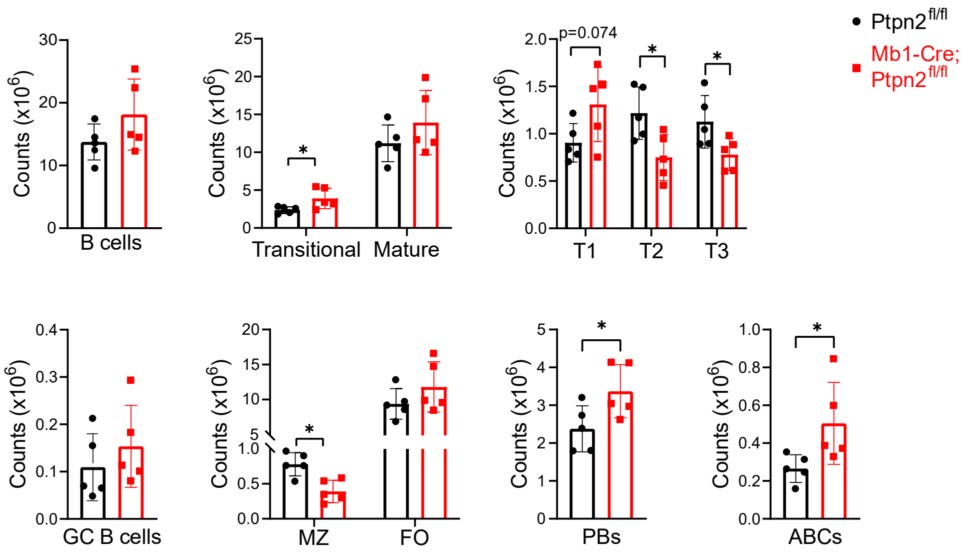

Supplemental Figure 7. Absolute cell counts of splenic B cell subset in *Ptpn2*-deficient mice. Absolute numbers of each B cell subset from 24 week old mice corresponding to populations shown in Figure 3 (n=5, *P < 0.05, 2-way ANOVA, Student's T-test).

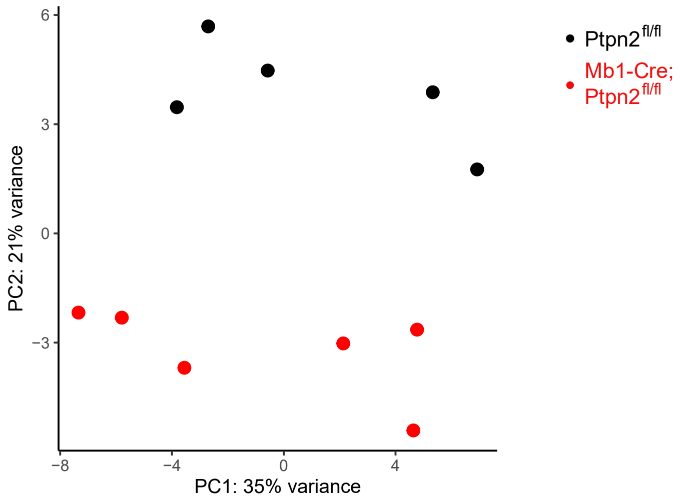

Supplemental Figure 8. Bulk RNAseq. PCA plot differentiated by genotype. Each point represents a sample, and colors indicated the respective genotypes (black = Ptpn2$^{fl/fl}$, red = Mb1-cre;Ptpn2$^{fl/fl}$, n=5-6).

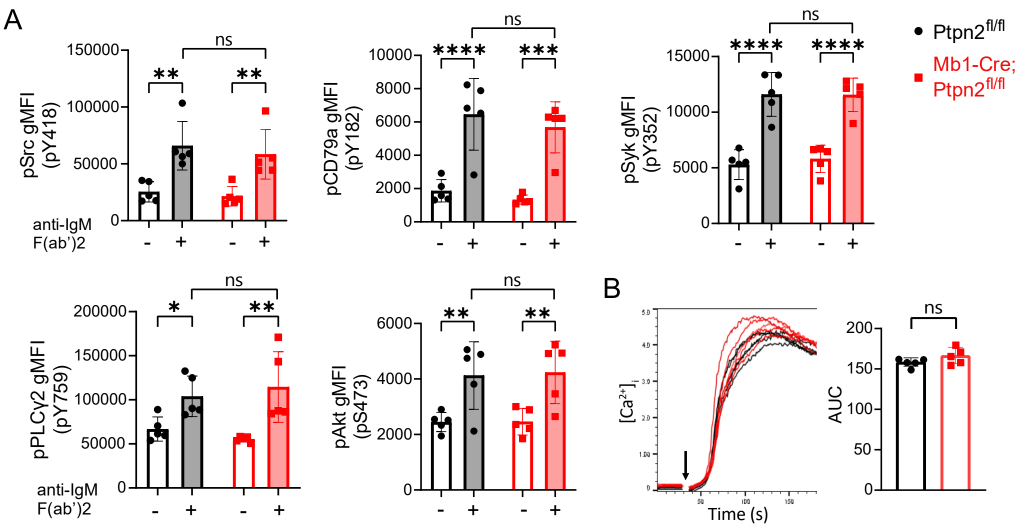

Supplemental Figure 9. B cell-specific *Ptpn2* deficiency does not affect BCR signaling. In 24 week old mice **A.** Phosphorylation of BCR signaling proteins (Src pY418, CD79a pY182, Syk pY352, PLCγ2 pY759, AKT pS473) after 3 minutes of anti-IgM F(ab)'2 stimulation. Data points represent B220+ cells from different mice. **B.** Calcium flux following anti-IgM F(ab)'2 (arrow) stimulation; area under curve (AUC) is quantified to the right. Data shown is gated on B220+ cells with equal surface IgM expression. Data is shown as mean ± SEM and is representative of ≥3 independent experiments (n=5). (*P < 0.05, **P < 0.01, ***P < 0.001, ****P < 0.0001, 2-way ANOVA).

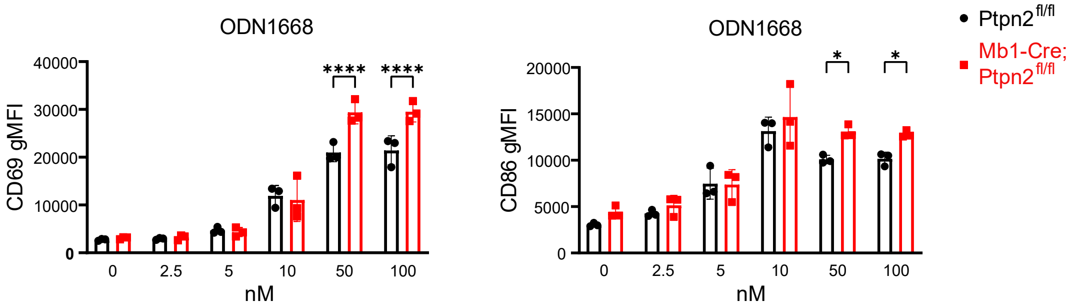

Supplemental Figure 10. TLR9 signaling increases in Mb1-cre;*Ptpn2*$^{fl/fl}$ mice. Representative data showing CD69 and CD86 expression after TLR9 (ODN1668) stimulation over dosage curve (0, 2.5, 5, 10, 25, 50, 100) (nM) in 24 week old mice (n=3). Data is shown as mean ± SEM and is representative of ≥3 independent experiments. (*P < 0.05, ****P < 0.0001, 2-way ANOVA).

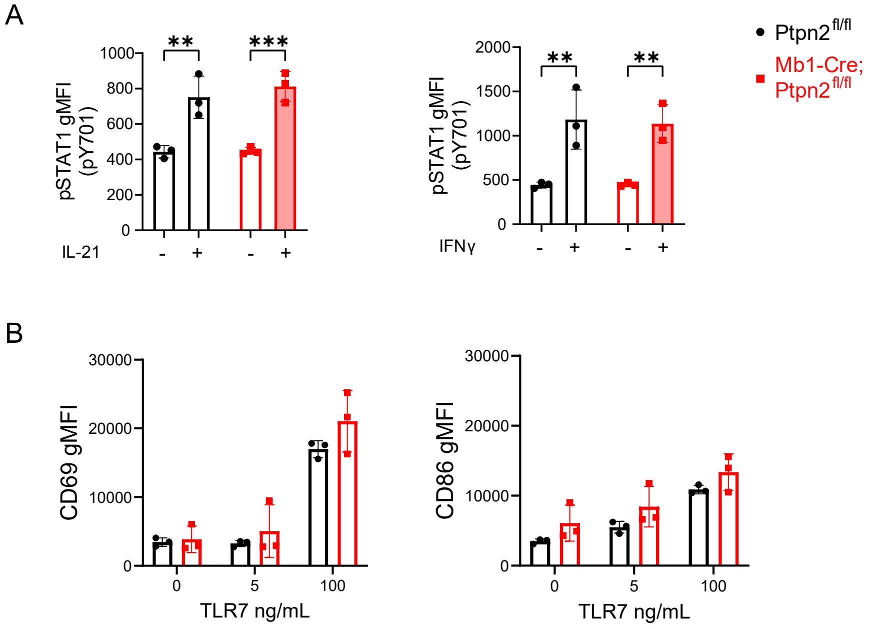

Supplemental Figure 11. B cell-specific *Ptpn2* deficiency does not affect JAK/STAT or TLR signaling in young mice. A. Phosphorylation of STAT1 after 10 minutes of IL-21 or IFNy- stimulation in 6-8 week old mice. **B.** CD69 and CD86 expression after TLR7 (R848) stimulation over dosage curve. Data points represent B220+ cells from different mice. Data is shown as mean ± SEM (n=3). (**P < 0.01, ***P < 0.001, 2-way ANOVA).

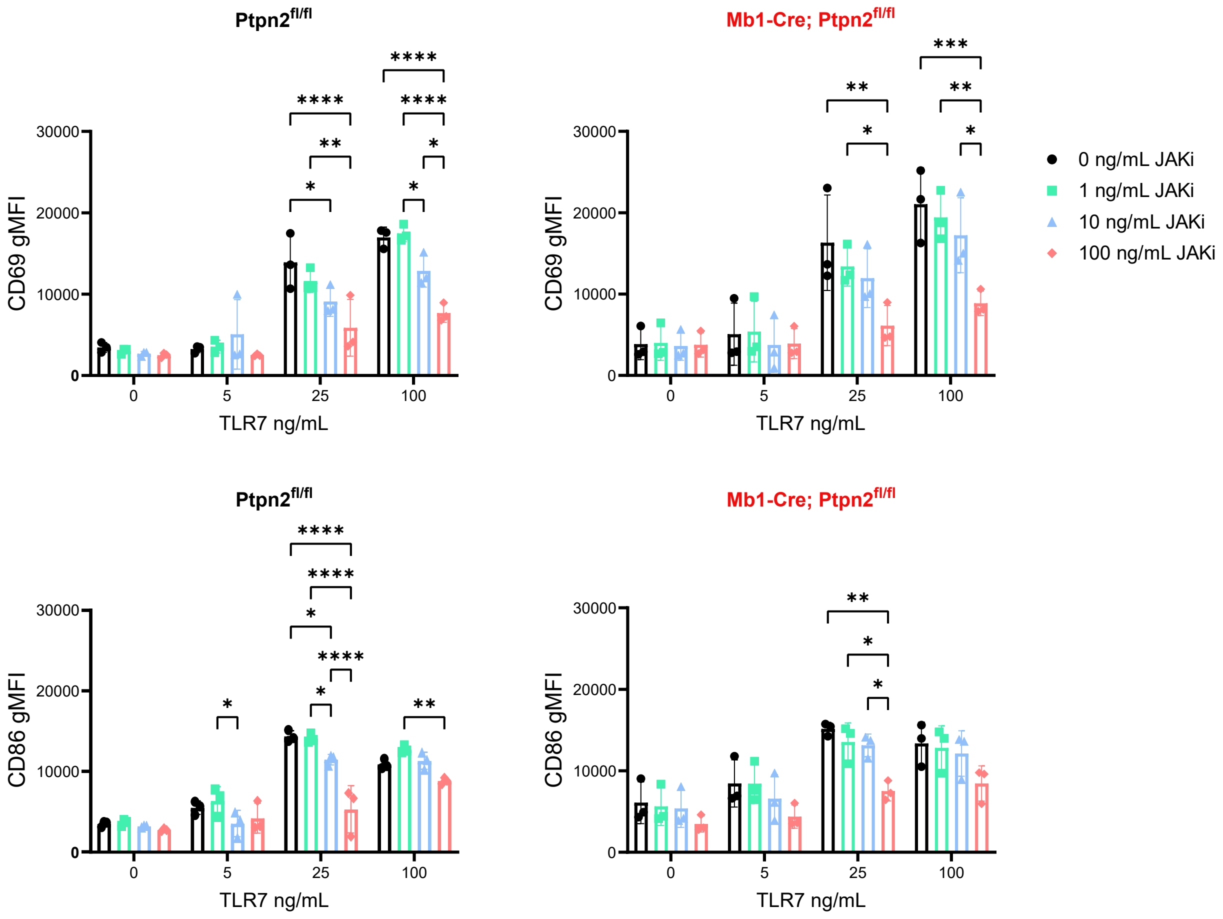

Supplemental Figure 12. JAK inhibition reduces TLR7-induced activation in *Ptpn2*$^{fl/fl}$ mice. CD69 and CD86 expression after TLR7 (R848) stimulation and JAK inhibition (Tofacitinib) over dosage curve (nM) (n=3) in 6-8 week old mice. Data is shown as mean ± SEM and is representative of 2 independent experiments (*P < 0.05, **P < 0.01, ***P < 0.001, ****P < 0.0001, 2-way ANOVA).
