# Supplementary figures and images for: Deletion of Ptpn2 in B cells promotes autoimmunity via TLR and JAK/STAT signaling
Source: JCI Insight. 2025 Dec 22;10(24):e196144. doi: 10.1172/jci.insight.196144 (PMC12890481; doi:10.1172/jci.insight.196144)

# Full unedited gels for Figure 1a

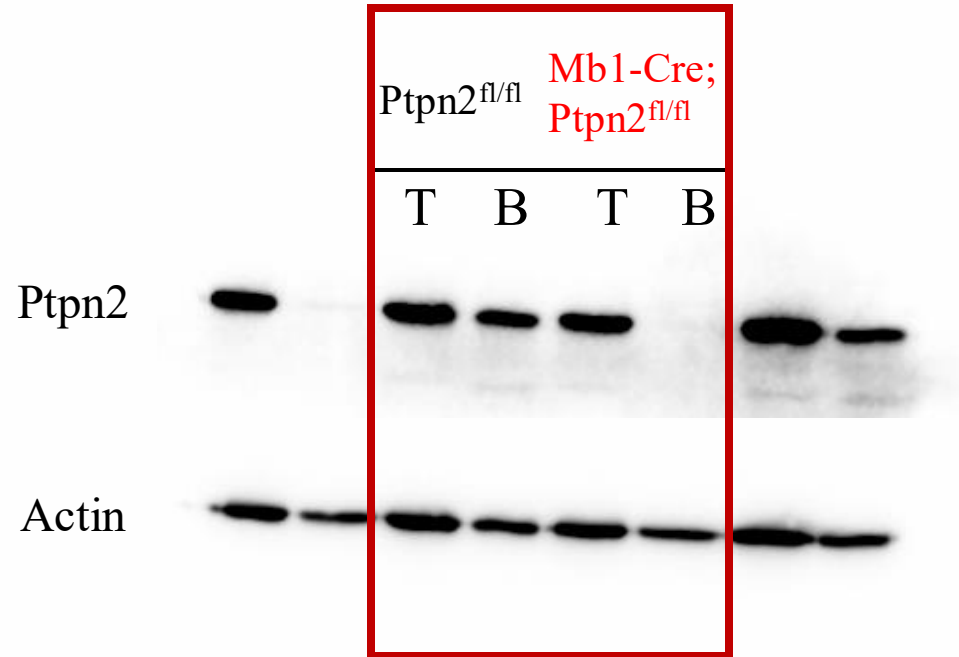

# Full unedited gels for Figure 6b

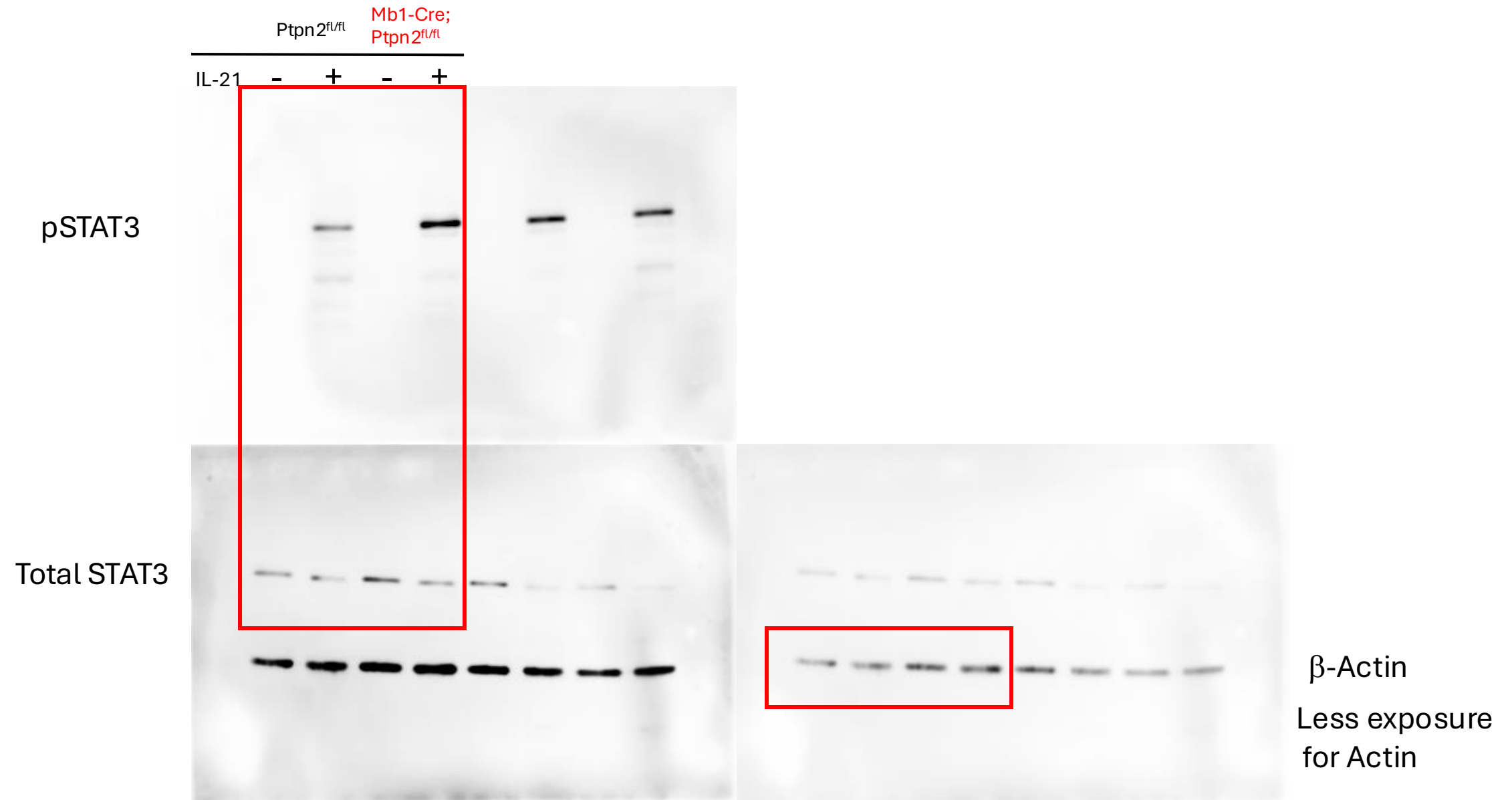

Supplement: Unedited blot and gel images [file jciinsight-10-196144-s097.pdf]
